# Current strategies for early epithelial ovarian cancer detection using miRNA as a potential tool

**DOI:** 10.3389/fmolb.2024.1361601

**Published:** 2024-04-16

**Authors:** Mridula Bhadra, Manisha Sachan, Seema Nara

**Affiliations:** Department of Biotechnology, Motilal Nehru National Institute of Technology-Allahabad, Prayagraj, Uttar Pradesh, India

**Keywords:** ovarian cancer, microRNA, biomarker, diagnosis, gynaecologic malignancy, aberrant expression

## Abstract

Ovarian cancer is one of the most aggressive and significant malignant tumor forms in the female reproductive system. It is the leading cause of death among gynecological cancers owing to its metastasis. Since its preliminary disease symptoms are lacking, it is imperative to develop early diagnostic biomarkers to aid in treatment optimization and personalization. In this vein, microRNAs, which are short sequence non-coding molecules, displayed great potential as highly specific and sensitive biomarker. miRNAs have been extensively advocated and proven to serve an instrumental part in the clinical management of cancer, especially ovarian cancer, by promoting the cancer cell progression, invasion, delayed apoptosis, epithelial-mesenchymal transition, metastasis of cancer cells, chemosensitivity and resistance and disease therapy. Here, we cover our present comprehension of the most up-to-date microRNA-based approaches to detect ovarian cancer, as well as current diagnostic and treatment strategies, the role of microRNAs as oncogenes or tumor suppressor genes, and their significance in ovarian cancer progression, prognosis, and therapy.

## 1 Introduction

According to a report by ICMR in 2019, ovarian cancer (OC) is among the top three most common cancers found among Indian women and 8th common cancer globally. It causes a higher mortality rate in women than any other type of reproductive cancer by far. Based on cancer stat facts by the National Cancer Institute for Ovarian Cancer the estimated new cases in 2023 were around 19,700 in the US itself, with estimated deaths of approximately 13,270. In accordance with its data of 2017–2019, it was approximated that, at some point in their lifetime, about 1.1 percent of women would be diagnosed with ovarian cancer. Based on the report, the 5-year survival rate decreased from 92% to 31% when the disease was screened at an advanced stage and the cancer metastasized to distant locations of the body (Author Anonymous, 2023). Women who have a family history of affected ovarian cancer, breast cancer, or both have an increased risk of encountering ovarian cancer at some point in their lives (Cannistra, 2004). World Health Organization (WHO) classified ovarian cancer into five categories: epithelial, germ cell, sex cord stromal, mesenchymal and rare ones under the miscellaneous category. The same cancer can also have different subtypes, such as low-grade and high-grade serous carcinoma (HGSC) (World Health Organization classification, 2023). The first three ovarian cancers are the primary ones, amongst which germ cell and sex cord stromal makeup roughly 5% (Stewart et al., 2019), whereas epithelial ovarian cancer (EOC) is the most commonly reported one. Sex cord stromal tumors are often symptomatic with signs such as hormonal production leading to menstrual changes and precocious puberty and has been reported to be associated with mutations in proteins such as STK11, DICER1 and FOXL2. Patients with sex cord stromal tumour currently have no peculiar treatment; instead, their care is similar to those with germ cell or epithelial tumour (Gershenson, 1994; Schultz et al., 2016). Germ cell tumors originate from germinal epithelium, primarily from primitive germ cells found in the developing ovary and constitute 2.6% of all ovarian tumors (Gică et al., 2022). It has been discovered that the β subunit of Human chorionic gonadotropin protein is one of the potent markers for ovarian germ cell tumour detection (Guo et al., 2011). Sonography, CT scans along with tumour markers like AFP, LDH and CA-125 are being used for the initial detection of germ cell tumors (Low et al., 2012).

EOC constitutes more than 90% of ovarian carcinoma and is further broadly categorized as slow-growing tumor Type-I and aggressive Type-II tumors based on mutation patterns present in the gene sequences. In the former, precursor lesions in the ovary are clearly visible, whereas in the latter, lesions are described to be formed *de novo* from the ovary surface epithelium cells. Low-grade serous, clear cell, mucinous, endometrioid, and transitional cell carcinomas are examples of type I epithelial ovarian tumors. In contrast, high-grade serous carcinoma and undifferentiated tumor are examples of type II epithelial ovarian tumors (Koshiyama et al., 2014). In many cases, ovarian endometrioid carcinomas are associated with mutations of the tumour suppressor phosphatase and tensin homolog deleted from chromosome 10 (PTEN). Some endometrioid-specific mutation patterns in the beta-catenin gene (CTNNB1) have also been found among 30% cases, which is rare to other subtypes (Obata et al., 1998; Palacios and Gamallo, 1998). More than half of high-grade serous carcinoma cases have been found to be mutated with tumor protein 53 (TP53) whereas mutations in KRAS (Kirsten rat sarcoma 2 viral oncogene homolog) or BRAF (v-raf murine sarcoma viral oncogene homolog) proteins are associated with low-grade serous carcinoma (Cho and Shih, 2009). For a more thorough analysis of mutation patterns observed in type-I and type-II carcinoma, study by Koshiyama et al. can be referred (Koshiyama et al., 2014). Compared to type II EOC, type I subgroup tumors are typically greater in size and are more frequently localized in the pelvis, more specifically limited to the ovaries themselves. As a result, type I tumors are easier to spot early using conventional methods as opposed to type II EOC, which tends to be found at advanced stages (stages III and IV), causing treatment challenging (Yemelyanova et al., 2008; Webb and Jordan, 2017; Charkhchi et al., 2020). While the best therapy is still an option, the design and development of upgraded screening methods focused on type II tumors may be able to identify aggressive malignancies.

The vague symptoms of ovarian cancer make it difficult to detect the onset of the disease. Till the time ovarian cancer is detected with nonspecific symptoms, such as abdominal bloating, pelvic and abdominal pain, enlarged abdominal size, frequent urination, having trouble eating and feeling full quickly, in some women, extreme weight loss (Goff, 2012), the malignancy has spread to the other pelvic regions of the body. At this point, treatment is highly challenging and is seen as a decrease in survival rate among the women diagnosed with this deadly disease.

There is currently no reliable screening technique with high specificity and sensitivity to identify early-stage OC. The dearth of identifiable symptoms in the initial phase of ovarian cancer, late detection and diagnosis, and the development of chemoresistance in cancer cells makes it difficult to plan and design an effective treatment for the disease causing a high mortality rate (Pal et al., 2015). On the basis of symptoms, some physical examinations are performed to examine the pelvis area and diagnose EOC, which includes transvaginal ultrasonography (TVS), and computed tomography (CT) [2]. Other conventional diagnostic methods include studying the history of disease, positron emission tomography-CT (PET-CT), magnetic resonance imaging (MRI) and ultrasonography. Merely 30%–45% of women with early-stage cancer can be identified using protein biomarker cancer antigen-125 (CA-125) and TVS (Wang et al., 2014a).

The imaging techniques discussed are frequently used to diagnose patients, but these procedures, however, use high radiation levels that could harm delicate tissues and cells, resulting in severe consequences for the patients. Also, despite being the primary treatments for ovarian cancer, surgery and chemotherapy have adverse effects that patients may experience. Consequently, identifying and diagnosing significant prognostic markers in women with OC at an early stage is one of the most significant strategies to increase their chance of survival. Early diagnosis may be achieved via novel detection techniques that can speed up and improve the accuracy of the test by analyzing the dysregulated expression of biomarkers in biological fluids. Micro-ribonucleic acids (miRNAs) have recently received attention in the context of OC because these present new approaches to screening, early detection, prevention, and therapy.

This study provides a thorough analysis of contemporary miRNA detection techniques in the context of ovarian cancer. It introduces the epidemiology, prognosis, treatment, and biomarkers of ovarian cancer before exploring both traditional and cutting-edge methods for diagnosing the disease. Additionally, the prospects for integrated platforms in the future to improve ovarian cancer diagnosis are explored. A thorough evaluation of all the most recent methods for identifying miRNAs associated with the early and precise diagnosis of OC is offered in this review paper.

## 2 Epithelial ovarian cancer: biomarkers and prediction strategies

Biomarkers are the biomolecules that are used to distinguish between a diseased state and a normal state by their irregular absence or presence or aberrant expression in the body. Its presence gives reliable data about the disease diagnosis and prognosis. The deficiency of symptoms during the early stage, along with ovarian cancer heterogeneity, often leads to poor diagnosis, delayed treatment, and development of distant metastases, resulting in high mortality rates among women. It presses on the need of exploring reliable ovarian cancer biomarkers that can be utilized for developing novel diagnostic solutions. Presently, apart from conventional biomarkers, multivariate assays and new circulating biomarkers are being used or explored for ovarian cancer diagnosis, prognosis or targeted therapeutics (Table 1). This section discusses some of the representative biomarkers in different categories.

**TABLE 1:** Different biomarkers explored for ovarian cancer management.

Biomarker	Type of biomarker	Source	Clinical significance	Status in OC	References
CA-125	Mucin-type glycoprotein	Plasma	Diagnostic	High expression	Dochez et al. (2019), Tripathi et al. (2020b), Hasenburg et al. (2021)
Human Epididymis Protein 4 (HE-4)	Protein (WDFC family) with a role of proteinase inhibitor	Serum	Diagnostic/prognostic	High expression	Rastogi et al. (2016), Zheng et al. (2018), Dochez et al. (2019), Navyatha and Nara (2019)
Mesothelin	Glycoprotein	Serum	Therapeutic	High expression	Rastogi et al. (2016), Ghafoor et al. (2018), Hilliard (2018)
Transthyretin	Prealbumin	Serum	Diagnostic	Low expression	Rastogi et al. (2016)
Osteopontin	Protein	Serum/ascites	Prognostic/diagnostic	High expression	Hu et al. (2015), Cerne et al. (2019)
Kallikreins	Protein (Serine protease)	Tissue/serum	Prognostic	High expression	Hibbs et al. (2004), Shan et al. (2006), Tamir et al. (2014)
Prostasin	Protein (Serine protease)	Serum	Diagnostic	High expression	Mok et al. (2001), Costa et al. (2009)
Lysophosphatidic acid (LPA)	Phospholipid	Serum	Prognostic	High expression	Murph et al. (2009), Li et al. (2015), Klymenko et al. (2020)
Transferrin	Glycoprotein	Serum	Diagnostic	Low expression	Macuks et al. (2010), Ivanova et al. (2022)
Aldehyde Dehydrogenase 1 (ALDH1)	Enzyme		Therapeutic	High expression	Xia et al. (2023)
Circulating tumor DNA (ctDNA)	Nucleic acid	Serum	Prognostic/diagnostic	High expression	Yang et al. (2021)
Micro RNA (miRNA)	Nucleic acid	Tissue/serum	Prognostic/diagnostic	High/low expression	Staicu et al. (2020)
Long non-coding RNA (lncRNA)	Nucleic acid		Therapeutic	High/low expression	Zhan et al. (2018), Luo et al. (2021), Beg et al. (2022)
Circulating RNA (CircRNAs)	Nucleic acid	Tissue/plasma	Therapeutic/diagnostic/prognostic	High/low expression	Qiu et al. (2022)
Bikunin	Glycosylated protein and a protease inhibitor	Serum/plasma/tissue	Prognostic	Low expression	Kobayashi et al. (2003), Tanaka et al. (2004), Matsuzaki et al. (2005)
Vascular Endothelial Growth Factor (VEGF)	Protein	Tissue/serum	Prognostic	High expression	Shen et al. (2000)
Apolipoprotein A-I (apoA-I)	High-density lipoprotein (HDL)	Serum	Diagnostic	Low expression	Rastogi et al. (2016), Atallah et al. (2021)
CA 19.9	Surface glycoprotein	Serum	Diagnostic	High expression	Tamakoshi et al. (1996), Kelly et al. (2010), Lee et al. (2020), Lertkhachonsuk et al. (2020)
Carcino-embryonic antigen (CEA)	Glycoprotein	Serum	Diagnostic/Prognostic	High expression	Guo et al. (2017), Lin et al. (2020), Wan et al. (2021), Kankanala and Mukkamalla (2023)

### 2.1 Conventional protein biomarkers

#### 2.1.1 Carbohydrate antigen (CA-125)

Among other biomarkers, serum CA-125, a glycoprotein of ∼200 to 5,000 kDa, has been employed routinely and is approved by the FDA for early epithelial ovarian cancer detection (Tripathi et al., 2020a). CA-125 was first defined by Bast et al. in 1981 using a monoclonal antibody (OC125) and was shown to be highly elevated when compared women with EOC to healthy controls (Bast et al., 1981). Although it has many benefits, CA-125 cannot be used to detect EOC in its early stages because of its low specificity and less adequate accuracy in women with ovarian cancer. Its levels are found to be increased in several other benign diseases, notably pelvic inflammatory disease, endometriosis, pregnancy, tuberculosis, cirrhosis of the liver and other non-gynecologic cancers such as lung and breast (Bast et al., 1998; Meden and Fattahi-Meibodi, 1998). Despite these limitations, CA-125 is regarded as one of the best ovarian cancer serum biomarkers currently accessible.

#### 2.1.2 Human epididymis protein 4 (HE-4)

Besides CA-125, HE-4 has been proven to be a biomarker with promising results in ovarian cancer screening. When CA-125 and HE-4 diagnostic performances in women with Type-I and Type-II ovarian cysts were compared, their combination showed the best diagnostic efficiency with the area under the curve value of 0.79 and 0.93 for Type-I and Type-II EOC, respectively (Kristjansdottir et al., 2013). In another research conducted by Fujiwara et al., in 2015, when assessed serum biomarker levels of CA-125 and HE-4 in 225 Japanese women with ovarian cancer and 94 healthy controls, a sensitivity of 92.1% in type-I and 78.8% for type-II were recorded when tested together. Compared to CA-125 and HE-4 alone, ROMA’s type I and type II sensitivities (84.8% and 97.4%, respectively) performed better. The findings of this research study have led to the conclusion that a more accurate way to differentiate patients with EOC from those with benign carcinoma is to evaluate HE-4 and CA-125 together with ROMA analysis instead of measuring either factor individually (Fujiwara et al., 2015).

### 2.2 Biomarker-based multivariate index assays (MIAs) and two-stage strategies for OC prediction

Multivariate assays were developed in response to the restricted specificity of single serum protein biomarkers in early detection of ovarian cancer. These assays incorporated multiple indexes to enhance the applicability of biomarkers.

#### 2.2.1 Risk of ovarian cancer algorithm (ROCA)

ROCA assay calculates the risk of occurrence of ovarian cancer based on gradual alteration in the CA-125 level over time (Gentry-Maharaj et al., 2020). This screening algorithm predicts the intermediate and elevated risk of ovarian cancer in women based on their age. Women with an average risk of occurrence are tested for the CA-125 level annually, whereas an intermediate risk calls for a test in every 3–4 months. A transvaginal scan (TVS) is referred to when there is an elevated risk of ROCA (Skates, 2012). Using data from the UK Collaborative Trial of Ovarian Cancer Screening (UKCTOCS), the Markov model of this study predicted a reduced mortality of 10% in contrast to the 11% found in the UKCTOCS trial. The screening has marginally improved ovarian cancer mortality but at great financial expense (Naumann and Brown, 2018).

#### 2.2.2 Risk of Malignancy Index (RMI) assay

Defined by Jacobs et al., in 1990, the formula used to define RMI was calculated as RMI = U x M x serum CA-125, in which M and U represent menopausal status and ultrasound results, respectively (Jacobs et al., 1990).

Later, in 2014, Javdekar and Maitra employed the Risk of Malignancy Index 2 (RMI 2) described by Tingulstad et al., value to distinguish between benign and malignant adnexal masses within a cohort of 58 women and found that RMI >200 had 70.5% sensitivity and 87.8% specificity (Tingulstad et al., 1999; Javdekar and Maitra, 2015).

#### 2.2.3 Risk of ovarian malignancy algorithm (ROMA) assay

Combining CA-125 and HE-4 levels, a multimarker assay was established by Moore et al. to foretell OC in women with a pelvic mass. The study included 566 women who were above the age of eighteen and had been diagnosed with an ovarian cyst or a pelvic tumor that would require surgery. Using the specified prediction probability criteria for premenopausal and postmenopausal women, this dual marker algorithm grouped individuals into high and low-risk malignancy groups (Moore et al., 2009).

#### 2.2.4 OVA1 assay

The first MIA to be granted approval in the U.S. by the U.S. Food and Drug Administration (FDA) in September 2009, this serum-based screening test predicts ovarian cancer malignancy by integrating the measured levels of five different proteins: transferrin, β-2 microglobulin, apolipoprotein A1, transthyretin, and CA 125-II. Based on menopausal status, an Ova 1 score is assigned, which designates a high malignancy when more than 5 or 4.4 in premenopausal and postmenopausal women, respectively (Muller, 2010; Ueland, 2017). In addition to the protein biomarkers previously mentioned, Ova-1 comprises menopausal status and imaging data (Jordan and Bristow, 2013).

### 2.3 International ovarian tumor analysis (IOTA)

Based on an ultrasound scan, the International Ovarian Tumor Analysis (IOTA) basic guidelines were established with a reported 92% sensitivity and 96% specificity. The IOTA group initially released a consensus article on words and criteria to characterize adnexal lesions in 2000 in an effort to improve diagnostic accuracy by homogenizing and standardizing the accuracy and assessment of ultrasonography across different centres (Timmerman et al., 2000). Based on the existence or lack of characteristic ultrasound characteristics of malignancy (such as ascites, enhanced vascularization, solid components, tumor size, papillary projections, and uneven cyst walls), IOTA classifies adnexal tumors (Kaijser et al., 2014). The study concluded that the best diagnostic tools currently available for determining whether an adnexal mass is benign or malignant in a preoperative scenario are the IOTA Logistic Regression Model (LR2) and Simple Rules.

### 2.4 Other predictive biomarkers for ovarian cancer detection

#### 2.4.1 Circulating tumor DNA (ctDNA)

In the 1970s, the first reports of circulating tumor DNA (ctDNA) being found in cancer patients’ blood appeared by Leon et al., which is now considered the most effective biomarker for OC. ctDNAs get actively released into the blood by cancer cells or apoptotic or necrotic cells. Since it reflects the genetic constitution of the cancer cell, it can be well exploited for examining the molecular makeup of the disease (Leon et al., 1977). Hou et al. investigated the prospective applications of circulating tumor DNA (ctDNA) as a prognostic biomarker for epithelial ovarian cancer by evaluating the correlation between ctDNA and CA-125 glycoprotein, pre-and post-treatment levels. The study showed that circulating tumor DNA strongly predicted cancer disease relapse, whereas the presence of CA-125 was not that effective for the same (Hou et al., 2022). Recently, Chen et al. developed a sensitive electrochemical biosensor for detecting DNA methylation in blood. The study detected DNA methylation in ovarian cancer patient blood samples and reported a detection limit of 2 aM of 110 nucleotide methylated DNA with single-site methylation (Chen et al., 2022).

### 2.5 DNA methylation

The process of adding methyl groups to the cytosine nucleotide is known as DNA methylation. A methyl group is typically inserted into a CpG site, which is a cytosine followed by a guanine. The most significant contribution of aberrant DNA methylation in OC progression has been shown to be associated with chemoresistance caused due to methylation at the promoter region of cancer resistance-associated genes. Song and Artibani reviewed the role of DNA methylation in OC- associated chemoresistance and methylation on ABC transporters. It was found to promote chemotherapeutic drug efflux, whereas downregulation in the expression of proapoptotic genes such as RASSF1A, MLH1 and MSH2 due to hypermethylation caused chemoresistance in cancer tissue samples (Song and Artibani, 2023). Another study in 2022 (Bisht et al., 2022) discussed the role of 5-hydroxymethylcytosine (5hmC), a demethylation intermediate, in OC. It was reported that elevated levels of 5hmC offer a favorable signal to prevent the growth and metastasis of cancer cells, improving the response to cancer treatment.

### 2.6 Tumor antigen associated autoantibodies

Tumor antigen-associated autoantibodies (AAb) are a reliable source of putative early diagnostic biomarkers for ovarian cancer, which are produced against mutated or overexpressed proteins in patients. Making use of custom protein microarrays, Anderson and team discovered a panel of 12 autoantibody biomarkers during clinical diagnosis in the sera of women with serous ovarian cancer but not in healthy women. Also, three potential autoantibodies of PTGFR, p53 and PTPRA with an AUC value of >60% (*p* < 0.01) were analyzed in the serum of OC patients (Anderson et al., 2015).

## 3 MicroRNAs: future biomarker for ovarian cancer management

MicroRNAs (miRNAs) are endogenously expressed molecules which are produced as long primary nucleic acid molecules (pri miRNAs) and are processed into precursor miRNAs (pre miRNAs) of ∼70 nucleotides. Pre-miRNAs are then transported outside the cell cytoplasm, and finally processed and cleaved to form mature miRNAs consisting of 18–24 nucleotides (Kim, 2005). These bind to the 3′ UTR of messenger RNAs and cause their functional repression or may lead to their direct degradation. Thus, they are also known for their negative regulation of gene expression (Zheng et al., 2013a). When it comes to cancers, miRNAs can function as tumour suppressors, slowing down the tumorigenesis process and its loss of function.

On the other hand, mutations in miRNAs may promote tumor formation or activate an oncogene. Oncogenic miRNAs, also called oncomirs, are primarily linked to cell proliferation and invasion, leading to tumor formation. Their expression level is seen to be highly upregulated in cancer tissues and is seen to stimulate metastasis of cancerous cells.

### 3.1 Significance of aberrant miRNA expression in ovarian cancer

Various molecular and functional studies have been done on *in vivo* and *in vitro* models demonstrated that aberrant miRNA expression is a critical regulator of ovarian cancer development. While there are not many instances of miRNA mutations during the progression of OC, their deregulated expression helps to understand the underlying pathophysiological state of the disease. It rendered them significant as a screening, diagnostic, therapeutic and prognostic biomarker for ovarian cancer as documented by several research groups. One such study by Braicu et al. (2017) identified overexpression of miR-325 in formalin-fixed paraffin-embedded ovarian cancer tissue samples and was shown to have a potential role in cancer cell invasion and metastasis, angiogenesis and promoting tumor progression. Another insightful investigation by Vang et al. (2013) reported differential expression of 17 miRNAs in omental lesions of ovarian tumor versus primary tumor. Among these 17 miRNAs, higher expression of miR-146a and miR-150 was found in omental lesions, stimulating patient survival and increased resistance towards cisplatin drugs. Yet another intriguing research by Calura et al. (2013), in the same year identified deregulated miRNAs in clear cell and mucinous histotypic subtypes of EOC. Five-fold higher levels of miR-192 and miR-194 were shown by mucinous histotype, while the clear cell histotype displayed five-fold higher expression of miR-30a-5p and miR-30a-3p, later negatively regulating the expression of E2F3 thus decreasing EOC cell proliferation.

In addition, a number of clinical trials have been carried out to investigate the diagnostic and prognostic potential of miRNA signatures and exploit miRNA expression patterns in ovarian malignancies. For example, clinical trial NCT03738319, completed in 2023 by Li to study the diagnostic and prognostic potential of exosomal miRNAs and long non-coding RNAs (lncRNA) in patients suffering from high-grade serous ovarian cancer (HGSOC) versus benign gynecologic diseases in a total of 120 individuals by next-generation sequencing. Another significant clinical trial NCT05146505, completed in 2023 by Perrone, investigated the expression level of miRNAs in women with HGSOC, and the correlation between deregulated miRNA expression and clinico-pathological and molecular data was established. Clinical trial NCT02758652 by Auranen, started in 2016, aims to investigate the significance of miRNAs in the development of chemoresistance in EOC and the variation in its expression in the prediction of primary treatment response. The miRNA expression from tissue, plasma and urine analyzed using microarray correlated with progression-free overall survival and the primary treatment response by the patient. One more clinical trial by Centre Francois Baclesse (NCT01391351) in 107 individuals completed in 2016 was based on serum miRNAs expression profile to identify predictors for chemotherapeutic response, i.e., taxol and carboplatin in OC patients. The trial investigated the serum miRNA profile before the chemotherapy and also identified Single Nucleotide polymorphism in genes involved in chemotherapeutic drug metabolism. Similarly, Li, in their clinical trial NCT03742856, analyzed the multi-omics results and studied the altered expression of RNA, including miRNA, mRNA and lncRNA, between patients of epithelial ovarian cancer with different FIGO stages and pathological subtypes. The study aimed to investigate the invasiveness and tumorigenesis of EOC by whole exome sequencing and analysis of transcriptomics and metabolomics.

### 3.2 miRNA expression profiling as a potential diagnostic, prognostic and predictive tool for ovarian cancer

Considering an urgent need for reliable biomarkers with high sensitivity and specificity, various studies have looked at how miRNAs differ in their expression patterns in ovarian cancer patients’ tissues, ascitic fluids, blood serum and plasma, serum extracellular vesicles such as exosomes and normal equivalents as well, to find their potential diagnostic and prognostic significance as biomarkers (Tables 2, 3). The diagnostic potential of miRNAs is evaluated in the form of AUC (Area under the receiver operating characteristic curve) value where the ROC curve distinguishes the diseased state of the patient as positive or negative based on test results aiming to find the optimal cut-off value to determine diagnostic efficiency of the microRNA, whereas AUC determines the diagnostic accuracy of the assay and the larger the area under the ROC curve the better is its diagnostic potential (Nahm, 2022). For instance, Zheng et al. (2013b) reported higher expression of miR-205 and lower expression of let-7f with a sensitivity of 30.1% and 66.9% respectively, which, demonstrates their diagnostic significance. Another study correlated the expression of miRNA-21 with clinicopathological features of EOC and reported higher expression in FIGO III-IV stages as compared to FIGO I–II stages (Xu et al., 2013).

**TABLE 2:** miRNA expression profile in ovarian cancer diagnosis.

miRNAs with diagnostic significance in ovarian cancer				
microRNA	OC subtype	miRNA expression pattern in OC	Diagnostic significance	References
let-7f	EOC	Downregulated	let-7f: Sensitivity-66.9% & specificity- 84.2% for; miR-205: Sensitivity-30.1% and specificity-94.2%	Zheng et al. (2013b)
miR-205		Upregulated		
miR-320a	EOC	Upregulated	miR-320a negatively targeted tumor suppressor gene RASSF8, which eventually promoted EOC cell proliferation	Zhang et al. (2021a)
miR-205-5p, miR-145-5p, miR-10a-5p, miR-328-3p and miR-346	OC	Upregulated	Combined AUC values of these 5 miRNAs were 0.788 and 0.763 for training and validation phases, respectively	Wang et al. (2019a)
miR-1246	HGSOC	Upregulated	Sensitivity- 87% and specificity- 77% for miR-1246	Todeschini et al. (2017)
miR-122, let-7i-5p, miR-25-3p and miR-152-5p	SOC	Downregulated	-	Langhe et al. (2015)
miR-200a, miR-200b, miR-373 and miR-200c	EOC	Upregulated	Combined sensitivity of 0.882 and specificity of 0.90 for miR-200a, miR-200b and miR-200c	Meng et al. (2016)
miR-93, miR-141, miR-155, miR-429, miR-200c, miR-205 and miR-492	OC	Upregulated	AUC values were 0.8235, 0.8717, 0.7962, 0.9328, 0.8445, 0.9475 and 0.9244 for miR-93, miR-141, miR-155, miR-429, miR-200c, miR-205 and miR-492, respectively	Braicu et al. (2017)
miR-200c-3p and miR-221-3p	OC	Upregulated	miR-200c-3p: AUC-0.92 and miR-221-3p-0.78 and a combined AUC of 0.824	Oliveira et al. (2019)

**TABLE 3:** miRNA expression profile in ovarian cancer prognosis.

miRNAs with prognostic significance in ovarian cancer				
microRNA	OC subtype	miRNA expression pattern in OC	Prognostic significance	References
let-7b	HGSOC	Downregulated	Increased expression of let-7b was associated with poor survival rates in HGSOC	Tang et al. (2014)
let-7f	EOC	Downregulated	Decreased expression of plasma let-7f was significantly correlated with poor progression free survival in all OC patients particularly in stage III-IV patients	Zheng et al. (2013b)
miR-148a-3p, miR-101-3p, miR-320d, miR-361-5p, miR-320a, miR-99a-5p, miR-500a-3p andmiR-664a-3p	OC	Downregulated	Impacted event free survival (EFS) negatively and caused shorter EFS	Krasniqi et al. (2021)
miR-1271-5p and miR-574-3p	EOC	Downregulated	-	Wang et al. (2014a)
miR-15b-5p, miR-96-5p, miR-182-5p, miR-182-3p, miR-183-5p, miR-141-5p, miR-130b-5p and miR-135b-3p		Upregulated		
miR-200a, miR-200b and miR-200c	EOC	Upregulated	High expression of miR-200a, miR-200b and miR-200c were associated with poor survival in advanced FIGO stage and high tumor grade	Cao et al. (2014)
miR-200c	OC	-	High expression of miR-200c inhibited TUBB3 expression and results in a good prognosis when HuR was nuclear	Prislei et al. (2013)
miR-135a-3p	OC	Downregulated	Increased expression of miR-135a-3p showed increased progression-free survival (PFS)	Fukagawa et al. (2017)
Let-7i	SOC	Downregulated	Lower let-7i expression caused chemoresistance and shorter PFS in patients	Langhe et al. (2015)
miR-21	EOC	Upregulated	Higher expression of miR-21 was correlated with shorter overall survival (OS), advanced FIGO stage and high tumor grade	Xu et al. (2013)
miR-221	EOC	Upregulated	Elevated level of miR-221 was found to be associated with FIGO stage and tumour grade and shortened OS in multivariate survival analysis	Hong et al. (2013)
miR-200a, miR-200b, miR-373 and miR-200c	EOC	Upregulated	Higher serum concentrations of exosomal miR-373, miR-200b and miR-200c were associated with poor OS and increased level of miR-200c was associated with shorter disease-free survival	Meng et al. (2016)
miR-130a	EOC	Upregulated	Higher expression of miR-130a found in advanced FIGO stage and was associated with serous histology in EOC patients	Prahm et al. (2021)

Although plasma and blood miRNAs are the preferred source for clinical diagnosis and non-invasive assay development, there seems to be a gap between miRNA expression patterns in tissue and plasma samples, and it's not yet clear if this expression in corresponding diseased tissues is accurately reflected in the patient plasma. Suryawanshi et al. tried to establish a correlation between tissue and plasma miRNA expression profiles by NanoString technology. When analyzed among 6 pairs of endometriosis-associated ovarian cancer tissue and plasma samples, a distinct expression profile was found in these samples without any significant correlation. Similarly, miR-135b-5p was found to be slightly upregulated in ovarian cancer tissue samples when compared to control, whereas this miRNA was highly downregulated in the validation phase in ascitic fluid of cancerous versus normal women (Suryawanshi et al., 2013). Another miRNA, miR-204-5p, was significantly downregulated in tissue samples when analyzed, whereas in the validation phase, it was found to be highly upregulated in ascitic fluid than in normal plasma showing a discrepancy in miRNA expression results (Záveský et al., 2019). The disparity among miRNA expression outcomes necessitates the need for more studies investigating the expression profiling of circulating miRNAs as well as their corresponding tumor tissues.

### 3.3 Major miRNA families regulating OC diagnosis and prognosis

From the preceding section, it is evident that the miRNA-200 and let-7 families are the two prominent miRNA families governing the arena of ovarian cancer diagnosis and prognosis. The miR-200 family, which primarily comprises miR-200a, miR-200b, miR-200c, miR-141 and miR-429, has been of utmost importance as it displays unusually high levels of expression with substantial correlation in the development of OC. This family of microRNAs are both tumor-suppressive and oncogenic and regulate genes associated with EMT in cancer, cancer cell growth, migration and proliferation, cell apoptosis and invasion (Kandettu et al., 2022).

Likewise, another miRNA family found to be widely associated with OC is the miRNA let-7 family. Let-7 came to be recognized as the first known human miRNA. Considering their extreme heterogeneity, the let-7 family of miRNAs is known to have a wide range of roles in EOC patients. Among the second most popular family of miRNAs discussed for ovarian cancer, let-7 family miRNAs are shown to promote cell differentiation and apoptosis. In addition to the diagnostic and prognostic functions these miRNA families contribute to, plenty of other reports assess other intriguing functions of this family. The role of both these families in ovarian cancer as documented in literature, is summarized in Tables 4, 5.

**TABLE 4:** Role of miR-200 family in ovarian cancer.

microRNA	Role in OC	References
miR-200c	Targeted class III β -tubulin (TUBB3) and increased chemosensitivity to drugs	Cochrane et al. (2010)
miR-200c	Targeted ZEB1 and ZEB2 and suppressed anoikis resistance	Howe et al. (2011)
miR-200b/200c/429	Targeted β-tubulin isotypes I, IIa, and III	Leskelä et al. (2011)
miR-200a and miR-200c	Targeted ZEB1 and ZEB2 and caused upregulation of E-cadherin	Park et al. (2008)
miR-200c	Increased cell proliferation & colony formation but reduced migration & invasion and targeted DLC-1	Ibrahim et al. (2015)
miR-429, miR-200a, miR-200b, miR-200c and miR-141	Targeted ZEB1 and ZEB2	Bendoraite et al. (2010)
miR-200b and miR-200c	Regulated angiogenesis by targeting IL-8 and CXCL1	Pecot et al. (2013)
miR200a, miR200b, and miR200c	Targeted MMP3 and ZEB1/pSMAD3 and inhibited cell invasiveness and metastasis	Sun et al. (2014)
miR-200a, miR-141, miR-200b and miR-200c	Targeted BAP-1	Iorio et al. (2007)
miR-200c	Targeted DLC-1 and regulated cancer cell proliferation, invasion and migration	Ibrahim et al. (2015)
miR-200c, miR-141	Increased sensitivity to paclitaxel and carboplatin and regulated EMT in cancer cells	Brozovic et al. (2015)
miR-141, miR-200a	Targeted p38α MAPK and regulated the oxidative stress response by cancer cells, increased sensitivity to paclitaxel	Mateescu et al. (2011)
miR-200c	Targeted TUBB3	Prislei et al. (2013)
miR-200c	Targeted VEGFR2 and increased the radiosensitivity of cancer cells	Shi et al. (2013)
miR-200c	Targeted VEGFA, FLT1, IKKβ, KLF9, FBLN5, and TIMP2 regulating angiogenesis, EMT, cell proliferation, apoptosis, invasion and tumorigenesis	Panda et al. (2012)
miR-429	Induced MET and increased sensitivity to platinum-based therapy	Wang et al. (2014b)

**TABLE 5:** Role of microRNA let-7 family in ovarian cancer.

microRNA	Expression	Role in OC	References
let-7b	-	Targeted HOST2 and promoted tumor cell migration in EOC	Gao et al. (2015)
let-7b	Downregulated	Inhibited EZH2 expression in OC cells	Kuang et al. (2021)
let-7d-5p	Downregulated	Targeted HMGA1 and promoted chemosensitivity in OC cells	Chen et al. (2019)
let-7e	-	Targeted EZH2 and CCND1 and increased sensitivity to cisplatin	Cai et al. (2013)
let-7b and let-7c	-	Caused poor outcome after postsurgery treatment and led to cancer progression	Tang et al. (2014)
miR-99b/let-7e/miR-125a cluster	Overexpressed	Targeted AT-rich interaction domain 3A (ARID3A)	Ma et al. (2017)
let-7e	Downregulated	Activated BRCA1 and Rad51 expression and caused double strand break repair	Xiao et al. (2017)
Let-7i	Downregulated	Targeted TLR4 and caused shorter PFS	Langhe et al. (2015)
let-7a	-	Stimulated the expression of IGF-II and caused cancer progression and poor survival, increased paclitaxel resistance	Lu et al. (2011)
let-7g	Overexpressed	Targeted IMP-1 and MDR1 and increased sensitivity to Taxol and vinblastine	Boyerinas et al. (2012)
let-7i	-	Targeted H-RAS and HMGA2 and altered resistance towards platinum-based chemotherapy in cells	Yang et al. (2008)
miR-98-5p	-	Induced cisplatin resistance and targeted 3′-UTR of Dicer1	Wang et al. (2018a)
let-7a-3	-	Regulated the expression of insulin-like growth factor-II (IGF-II) and insulin-like growth factor binding protein-3 (IGFBP-3)	Lu et al. (2007)

### 3.4 miRNAs: as therapeutic molecule for ovarian cancer

As miRNAs aberrant expression is shown to have an impact on almost all pathways of carcinogenesis such as epithelial-mesenchymal transition, angiogenesis, changes in extracellular matrix biology with cancer cell proliferation, invasion and metastasis, response and chemosensitivity to drugs (Cochrane et al., 2010; Brozovic et al., 2015; Ibrahim et al., 2015; Wang et al., 2018; Ferreira et al., 2020), these are also being explored as a therapeutic molecule. Some recent reviews have highlighted the role of miRNAs in diagnosis, prognosis and designing therapeutic strategies for ovarian cancer (Davies et al., 2022; Zhao et al., 2022; Asl et al., 2023). A recent strategy employs synthetically produced RNA molecules known as RNA sponges as one of the emerging therapeutic approaches, which is designed to possess a number of sites for target miRNA binding with high affinity. With oncomiR silencing capability, miRNA sponges act as vital therapeutic agents by blocking one or multiple oncogenic miRNAs simultaneously. Another strategy to treat ovarian cancer through miRNA is by employing miRNA mimics, artificially synthesized RNA duplexes, to recompense the reduced expression of endogenous tumor-suppressing miRNAs by mimicking them (Ebert et al., 2007; Kluiver et al., 2012). In April 2013, the first miRNA mimic, MRX34, made its way to phase I clinical trial in order to treat patients with primary liver cancer (Austin, 2013). Nakano et al. transfected an ovarian cancer cell line with 319 miRNA mimics and observed a major decrease in cell proliferation (Nakano et al., 2013). By transfecting cells with miR-494 mimics, Yang and team investigated the role of miR-494 expression in EOC progression, cell proliferation, and metastasis (Yang et al., 2017). Concluding its tumor suppressive role in EOC via targeting sirtuin 1 (SIRT1), miR-494 was shown to have a promising role in EOC treatment. Another interesting observation by Gandham et al. added to therapeutic potential of miRNA mimics in treating ovarian cancer patients. Their study used nanoparticle formulation with hyaluronic acid to deliver Let-7b miRNA mimic into tumor cells. It concluded that a combination of Let-7b miRNA mimic and PTX l (paclitaxel) led to an enhanced anti-tumor efficacy as it improved the potency of PTX, decreasing the overall IC_50_ value to 13-fold (Gandham et al., 2022). Other potential strategies, such as miRNA masking and inhibition by small molecule miRNA inhibitors (SMIRs) have also been investigated as a possible therapeutic approach in which SMIRs can directly bind to target oncogenic miRNAs, masking subsequently its effect, whereas in miRNA masking, an oligonucleotide binds to the 3′UTR of the mRNA preventing its degradation by miRNA (Bader et al., 2010; Monroig et al., 2015; Barbu et al., 2020; Zhang et al., 2020). Vernon et al. stated that miRNA is an effective molecule for ovarian cancer chemotherapy, wherein, miR-3622b-5p induced OC cell apoptosis and decreased migration when combined with EGFR inhibitors by increasing the OC cell’s sensitivity to platinum drugs. It was also concluded that miRNAs could be therapeutically applied to treat OC by killing and real-time monitoring of drug-resistant OC cells (Vernon et al., 2020).

The present review mainly focuses on the technological aspects of miRNA detection, including the cutting-edge strategies and their associated challenges. But several other significant roles by miRNA such as in signaling pathways involved in OC, metastasis and drug sensitivity, miRNAs in predicting overall patient survival and the epigenetic regulation of miRNAs have been highlighted in some of the interesting reviews (Zhang et al., 2008; Wang et al., 2014; Pal et al., 2015; Ismail et al., 2022) which the readers can refer for more detailed insight on miRNA-based OC management.

### 3.5 Conventional miRNA detection methods

#### 3.5.1 Quantitative real-time polymerase chain reaction (qRT-PCR)

qRT-PCR is an amplification-based real-time expression analysis method for nucleic acids. On the basis of the cycle threshold (Ct) value and amplification plot, the miRNA expression is quantitated. It is a gold standard and one of the widely used methods for miRNA analysis. This method requires a smaller sample amount and is very sensitive and specific, but it is a contamination-prone method, necessitating extremely precise handling (Koshiol et al., 2010). Over the years, different forms of qRT-PCR has emerged, enabling extensive sensitivity to miRNA detection. qRT-PCR is a high throughput technique which can also be used to check the expression of precursor miRNAs (pre-miRNAs) (Schmittgen et al., 2004). The need to design individual target-specific fluorophore labelled probes makes the technique less cost effective and unsuitable for large amounts of miRNA analysis. Also, because the quality of the miRNA extract significantly impacts miRNA analysis using qRT-PCR, the results of miRNA analysis vary depending on the sampling technique used on the same sample. For example, Taylor et al. reported that a qPCR experiment could encounter errors associated with subsampling and biological variability, as well as technical errors related to the calibration of the equipment used (Taylor et al., 2019). In a recent study designed by Hu et al., graphene oxide (GO) nanoparticles were used to detect miRNA via qRT-PCR, which enhanced the specificity and sensitivity of the assay. Adding GO to the qPCR reaction assay significantly enhanced the PCR efficiency by minimizing the non-specific amplification and strengthening primer-template hybridization efficiency. Also, when compared to conventional qRT-PCR, the concentration of primers in the region where GO exists was comparatively higher due to GO-mediated adsorption of primers by π-π stacking, in turn increasing the efficiency of primer-template hybridization during annealing (Hu et al., 2021).

#### 3.5.2 Northern blotting

Northern blotting, which is based on molecular hybridization and gel electrophoresis, performs quantitative miRNA analysis. Typically, in a Northern blot, RNAs with different molecular weights and lengths are run under an electric field, separated by gel electrophoresis, and then detected using a fluorophore-tagged probe that hybridizes with the target RNA. This method of RNA estimation does not require any preliminary processing of RNA and makes the process reliable due to no change to the nucleotide bases (Ouyang et al., 2019). Northern blotting can detect the size of mature RNAs and precursor RNAs (Koscianska et al., 2011). However, it has a few constraints-the process is labor-intensive and needs expensive reagents and a lot of starting material, making it less suitable for high throughput studies. This method is complex and has low throughput, which requires the use of radiolabeled oligonucleotide probes. (Koshiol et al., 2010). Other drawbacks of the northern blotting technique includes the use of carcinogenic chemicals such as formaldehyde, ethidium bromide and radioactive probes and the sensitivity of the sample towards RNAse degradation (Kilic et al., 2018).

#### 3.5.3 Microarray

Thanks to the potent high-throughput capabilities of microarray technology in which a single experiment may monitor the expression of thousands of short non-coding RNAs simultaneously in hundreds of samples processed (Liu et al., 2008). This method is high throughput and requires much less RNA as starting material. Although it is the most widely used technique for miRNA detection, the process is costly due to fabrication costs, and it may cause cross-hybridization between homologous miRNA sequences, reducing its sensitivity (Koshiol et al., 2010; Kilic et al., 2018). Including 428 patients with ovarian tumors, Yokoi et al. designed a microarray-based diagnostic model which had a 99% sensitivity and 100% specificity to check the expression levels of ten miRNAs which were further verified using qRT-PCR to increase the accuracy as well as the usefulness of the developed model (Yokoi et al., 2018).

#### 3.5.4 RNA sequencing

**FIGURE 1:**
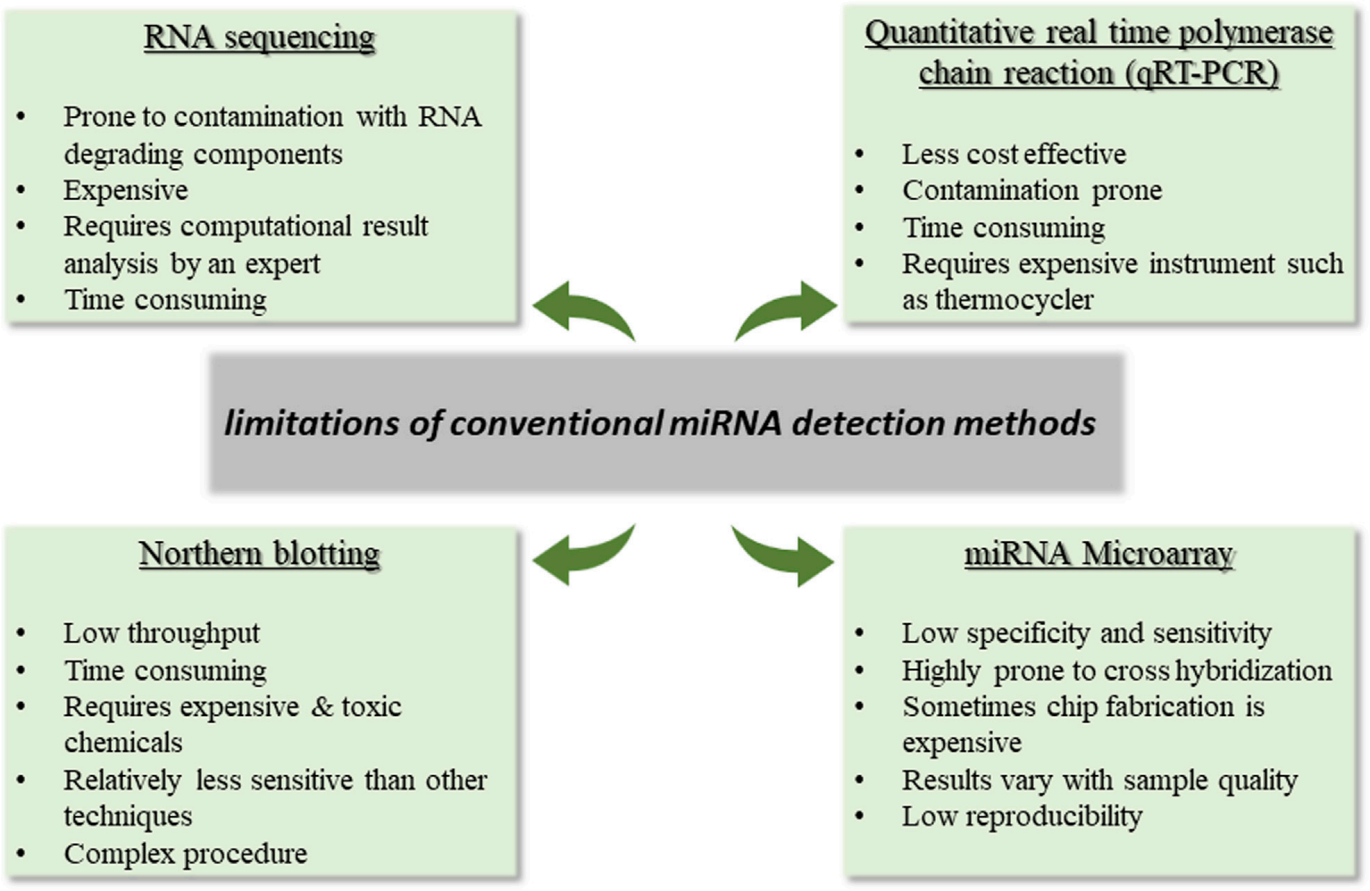
Depicts the limitations of conventional miRNA detection techniques.

For miRNA analysis, high throughput sequencing or next-generation sequencing (NGS) are frequently employed to obtain genetic information. Despite advancements in sequencing techniques, this approach needed to overcome certain limitations. For instance, as having a complex workflow, the technical strategies used for library preparation can impact the detection of microRNA by NGS by skewing the relative abundance of the chosen microRNAs across various samples. Shi and group have reported that when using RNA-seq technology, biases and errors can be introduced at different stages, including sample preparation, library building, sequencing, and imaging (Shi et al., 2021). A comparative analysis and limitations of the conventional miRNA detection methods are depicted in Figure 1 (Koshiol et al., 2010; Chugh and Dittmer, 2012; Hardikar et al., 2014; Islam et al., 2017; Dave et al., 2019).

## 4 Modern approaches for miRNA-mediated OC detection

Modern methods for ovarian cancer early detection are essential for appropriate medication and customized treatment. The following section is categorized into two sections-the first section discusses the computer-aided methods that integrate various studies on identifying the role of miRNAs in ovarian cancer diagnosis and prognosis based upon conventional miRNA detection techniques. In contrast, the second section emphasizes the modern technique-based approaches and strategies that has been in use lately for the miRNA-based OC detection.

### 4.1 Computer-aided miRNA detection approaches

#### 4.1.1 Meta analysis-based miRNA detection

Including studies that demonstrated a relationship between miRNA expression and EOC, a systematic review was done by Ferreira et al., in 2020, which included articles published before February 2019, in which twelve miRNAs were found to have prognostic and predictive value. miRNA features, such as their expression profile in OC tissue and serum and chemosensitivity to paclitaxel or platinum-based drugs were considered for the study. By analyzing their predictive response and prognostic features, their significance in cancer management was evaluated (Ferreira et al., 2020).

A meta-analysis of miRNA microarray by Wang et al. identified the significance of miR-27a in ovarian cancer cell proliferation and migration. It was observed that miR-27a can promote cancer progression by modulating FOXO1 expression (Wang et al., 2018). Another latest work conducted by Frisk and team done a systematic review and meta-analysis to identify potential circulating miRNAs for early diagnosis of OC. A total of 22 articles were taken into consideration for the study of the quantitative meta-analysis, among which nine miRNAs were found to have dysregulated expression in OC datasets, including miR-21, miR-125, miR-141, miR-145, miR-205, miR-328, miR-200a, miR-200b and miR-200c (Frisk et al., 2023). Meta-analytical studies identifying ovarian cancer based on dysregulated miRNA expression are tabulated in Table 6.

**TABLE 6:** Micro RNA-based meta-analysis studies on ovarian cancer.

Method for analysis/sample	Software for statistical analysis	miRNAs analyzed	Models used	Number of patients for the study	References
CENTRAL, MEDLINE, and EMBASE	OpenMetaAnalyst, STATA 14.0 and RevMan 5.3	miRNA 200a, miR-200b, miR-200c	Bivariate random-effects model	1,081 patients with ovarian cancer and 518 controls	Wang et al. (2019b)
Robust Rank Aggregation (RRA) method	-	-	Bivariate meta-analysis model	519 patients with epithelial ovarian cancer and 248 controls	Teng et al. (2016)
**-**	Meta-package in R studio	miR-21-5p, miR-26-5p, miR-93-5p, miR-106b-5p, miR-125b-5p, miR-141-5p, miR-145-5p, miR-205-5p, miR-200a-3p, miR-200b-3p, miR-200c-3p, miR-328-5p, and miR-429-3p	Fixed-effect model	3387 OC patients, 3461 healthy women, and 475 women with benign cysts	Frisk et al. (2023)
miRNA microarrays	-	miR-27a	Fixed effect model	-	Wang et al. (2018b)
**-**	Stata 14.0	-	Bivariate mixed-effect models	1,356 participants	Wang et al. (2017)
**-**	STATA 16.0	miR-145	Fixed-effects model and random-effects model	-	Chen et al. (2021)
**-**	STATA	12 miRNAs	Random effect model	-	Ferreira et al. (2020)
miRNA microarrays or sequencing	STATE 12.0	30 miRNAs	Bivariate model and random-effects model	3,470 patients with ovarian cancer and 1606 healthy controls	Zhou et al. (2018)
-	Stata 12.0	miR-205, miR-145, miR-429, miR-141, miR-125b, miR-200c, miR-200a and miR-200b	Random effects model and fixed effects model	1,485 patients with ovarian cancer and 1182 controls	Cui et al. (2020)
-	Stata 12.0	miR-200a, miR-200c miR-141 and miR-30d-5p	Random-effects model and fixed-effect model	1,596 patients with ovarian cancer	Shi et al. (2018)
miRNA microarrays	STATA 12.0	-	Random effects model	553 patients with ovarian cancer	Shi and Zhang (2016)
**-**	Meta Disc 1.4	11 miRNAs	Bivariate meta-analysis model, fixed effect models and Random effect model	1732 OC patients and 3958 controls	Zhang et al. (2021b)

#### 4.1.2 In silico-based miRNA detection

Similar to metanalysis, White et al. worked to understand the mechanism of post-transcriptional regulation of KLK gene expression and its regulation by miRNA, for which the expression of miRNAs in OC was analyzed *in silico*. A total of ninety-eight miRNAs were found to carry altered expression when studied *in silico* among which three miRNAs were expected to target KLK10 gene, which led to reduced cell proliferation and less cancer progression. miRNAs, miR-224, let-7f and miR-516a, were shown to successfully target the gene, causing reduced expression of KLK10 protein and less cellular growth, suggesting their efficient therapeutic application in OC treatment (White et al., 2010).

Combining bioinformatics and meta-analysis, Wu and team screened potential serum miRNAs for ovarian cancer diagnosis using *in silico* approach. Sequencing data from the Gene Expression Omnibus (GEO) database were selected, and differentially expressed miRNAs were profiled. Five miRNAs, miR-200a, miR-200b, miR-25, miR-200c and miR-429, were deregulated in ovarian tumor patients when compared to healthy controls with an integrated sensitivity and specificity of 64% & 88%, respectively, and AUC value of 0.78. At the same time, miR-200 family produced a combined AUC value of 0.78 with a sensitivity of 64% and specificity of 88% when analyzed using meta-analysis (Wu et al., 2019). Table 7 concludes *in silico* studies studying the significance of aberrantly expressed miRNAs on ovarian cancer patients.

**TABLE 7:** *In silico* studies to determine dysregulated miRNAs in ovarian cancer patients.

Databases used	miRNAs analyzed	Samples	References
GSE119055	miR-18a-5p, miR-130b-3p, miR-182-5p, miR-187-3p, miR-378a-3p, miR-500a-3p, miR-501-3p, miR-4324, miR-1271-5p, and miR-660-5p	6 OC patients and 3 healthy samples	Beg et al. (2023)
GSE83693, GSE119055 and GSE98391	miR-378a-3p, miR-378a-5p and miR-378c	Tissue samples from human and mouse ovarian cancer and normal cells	Mohammed et al. (2023)
GSE43867	miR-760, miR-483-5p, miR-766, miR-198, miR-129-3p and miR-642	86 SEOC patients	Wei et al. (2015)

#### 4.1.3 Machine learning-based miRNA detection

Hamidi and team identified circulating miRNAs as biomarkers to predict the occurrence of OC utilizing a machine learning approach. In this study, Boruta, random forest-based variable selection approach in machine-learning technique, and other statistical approaches were used, which reported ten distinguishing miRNA biomarkers, namely miR-1233-5p, miR-4675, miR-1290, miR-1914-5p, miR-1469, miR-6784-5p, miR-6800-5p, miR-3184-5p, miR-5100 and miR-1228-5p, with differential expression in ovarian cancer datasets than in normal cases (Hamidi et al., 2023). Another study used the Evolutionary Biomarker Search Tool (EBST), a novel technique based on gene expression data to discover microRNAs with biomarker potential in ovarian cancer (Yaghoobi et al., 2020). After pre-processing the data and mathematical validations, the proposed model identified 11 miRNAs as biomarkers including, miR-1228-5p, miR-8073, miR-6756-5p, miR-3663-3p, miR-4697-5p, miR-6784-5p, miR-1307-3p, miR-1228-3p, miR-328-5p, miR-6821-5p and miR-1268a. This model had 100% sensitivity and 99.38% specificity with an accuracy of 99.69%.

#### 4.1.4 Multi omics-based miRNA detection

Multi omics approach integrates various datasets based on transcriptomics, metabolomics, microarray, genomics and proteomics studies and is studied by biological scientists to extract the required information of a biological system or a cell and describe the disease profile. Analyzing miRNA data by multi omics approach based on miRNA sequencing, the Indian cohort’s HGSOC samples displayed significant expression levels of the miR-200 and miR-1269a families (Mhatre et al., 2023). Their results displayed population-specific gene expression patterns and molecular signatures based on the histotypes found in the Indian cohort. In a related work that focuses on the multi omics approach, by combining transcriptome data with genome-scale biomolecular networks, it was discovered that miR-16-5p expression and miR124-3p expression varied in ovarian cancer tissues and can act as potent biomarkers for ovarian cancer detection (Gov et al., 2017). Differential network mapping is often used to study molecules associated with disease occurrence and progression by investigating existing datasets.

### 4.2 Other modern strategies for miRNA detection

#### 4.2.1 Biosensor based miRNA detection

The current conventional methodologies for cancer biomarker detection quickly give way to sensor-based alternatives. Biosensors are compact, quick, extremely sensitive, and selective devices. A biosensor has two basic elements: the biorecognition part, which is responsible for recognizing and interacting with the target molecule, and the transducer part, which converts this interaction into readable signals (Ivanov et al., 2022). Extensive research is ongoing to develop miRNA-based sensors for disease detection, including cancers.

For instance, in a recent work by Ivanov et al., three ovarian cancer-associated plasma miRNA biomarkers, miRNA-21, miRNA-141, and miRNA-200a were detected using silicon nanowire-based biosensors (Ivanov et al., 2022). Here, oligonucleotide probes (oDNA probes) which are complementary to target miRNA, were immobilized on a silicon nanowire-modified sensor surface which detected the miRNAs with a sensitivity of 1.1 × 10^−16^ M. In this study, it was stated that being non-amplification-based method, this strategy was less prone to contamination, allowing high specificity in contrast to qPCR, which has sensitivity but is extremely prone to sample contamination. Similarly, in another study, Moazampour and team designed an electrochemical biosensor having surface modification with L-cysteine functionalized Zinc sulfide quantum dots with a detection range between 1.0 × 10^−14^ to 1.0 × 10^−6^ M for miR-200a, a potential ovarian cancer biomarker (Moazampour et al., 2021). QDs functionalized with L-cysteine allowed immobilization of DNA probe on the glassy carbon electrode (GCE) surface, which differentiated target miRNA-200a strand from a single-base mismatch in miRNA strand and confirmed using electrochemical impedance spectroscopy (EIS). This label-free genosensor with the surface functionalization provided an extra active site for easy capture of the probe upon EDC/NHS activation without the requirement of sample pretreatment and led to a sensitivity of 8.4 fM in contrast to other similar works. Table 8 summarizes research reports on miRNA-based biosensors for ovarian cancer detection. Another latest study by Islam et al. included graphene oxide-loaded iron oxide (GO/IO hybrid material) having RuHex [ruthenium hexammine (III) chloride (Ru (NH_3_)_6_]^3^, reducing and electrocatalytic activity for ultrasensitive detection of miR-21 up to 1.0 fM. Here, the screen-printed carbon electrode (SPCE) was modified with the hybrid nanomaterial, responsible for the specific adsorption of target miRNA to the sensor surface and producing a chronocoulometric signal (Islam et al., 2018).

**TABLE 8:** Micro RNA-based biosensors for ovarian cancer detection.

Biosensor type	Target miRNA	Detection range	Detection limit	References
Silicon-On-Insulator Nanowire	miRNA-21, miR-141, and miR-200a	-	1.1 × 10^−16^ M	Ivanov et al. (2022)
Colorimetric nano-biosensor	miRNA-21, miR-155	-	<1 ng/μL	Mollasalehi and Shajari (2021)
ZnS quantum dots electrochemical biosensor	miR-200a	1.0 × 10^−14^ to 1.0 × 10^−6^ M	8.4 fM	Moazampour et al. (2021)
Electrochemical biosensor	miRNA-21	-	1.0 fM	Islam et al. (2018)

#### 4.2.2 Microfluidics-based miRNA detection

**FIGURE 2:**
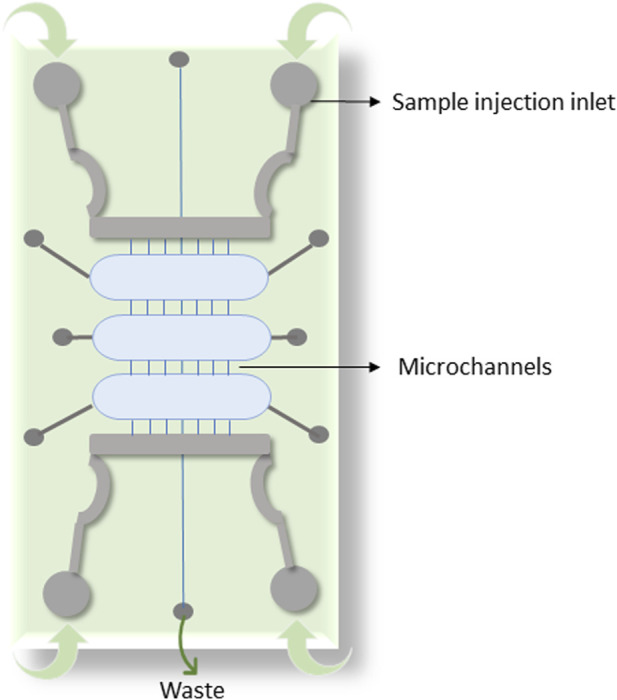
Depicts a basic representation of a microfluidic chip for miRNA detection.

Despite the availability of multiple miRNA detection techniques, a quicker and more precise method for diagnosis is still required and herein lies the application of microfluidic systems. Microfluidic devices have a controlled fluid flow comprised of small parts, now among the highly prominent and accepted platforms for cancer diagnosis, prognosis and research. A basic representation of microfluic system is shown in Figure 2. miRNA-based integrated microfluidic devices are among the most sensitive and user-friendly devices developed so far in modern medicine. Apart from being highly sensitive, these offer other advantages such as the requirement of less amount of sample, miniaturization, real-time output with less reaction time, and ease of use. To detect miRNA levels, optical or electrochemical biosensors are frequently coupled with microfluidic technologies.

By integrating a microfluidic platform with digital PCR (dPCR), Sung et al., quantified the amount of plasma miRNA-21 with a detection limit of 1.4 attomolar for ovarian cancer detection. This design showed an increased PCR efficiency with absolute miRNA quantification with no sample pretreatment and detection of the target at an extremely low concentration (Sung et al., 2021). A similar design by the same team was again reported in 2022, which depicted the quantification and detection of extracellular vesicle (EV) derived miR-21 and miR-200a by integrating multiplex digital PCR and microfluidic system for early screening and prognosis of ovarian cancer. Here, the entire process from EV extraction to multiplex dPCR was integrated on the microfluidic chip. The target miRNA was quantified in a brief amount of time—120 minutes—and the PCR calibration curve displayed a high linearity rate with no false positive signal for the simultaneous detection of both miRNAs. This design also had an EV capture rate that was 1.8 times higher. (Huang et al., 2022). Another study discussed various aptamer-based microfluidics systems for detecting ovarian cancer via targeting biomarkers such as CA-125 and CEA and ovarian cancer cells (Vandghanooni et al., 2021).

#### 4.2.3 Fluorescence based miRNA detection

A non-PCR based miRNA detection employing fluorescence has been widely used via a toehold-mediated strand displacement (TMSD) reaction for disease diagnosis, including cancers. It is driven by Gibb’s free energy, involving three steps, including target toehold binding with the invader strand, which is followed by branch migration and chain dissociation (Mayer et al., 2022). Recently, a TMSD-based method employing fluorescence has been developed by Sun et al., in 2023 to measure miRNA-21 and diagnose ovarian cancer early. The study developed an enzyme-free, rapid, non-PCR dependent, isothermal signal amplification assay with 6.05 pM as the lower detection limit. Furthermore, a strong linear correlation between the concentration of target microRNA and the strength of the fluorescence signal was found using quantitative analysis based on the change in fluorescence intensity at miR-21 concentrations ranging from 10 to 100 nM (Sun et al., 2023).

#### 4.2.4 CRISPR/Cas system-based miRNA detection

Another recent technological progress in miRNA detection includes the CRISPR/Cas system, which is an amplification-free approach which employs liposome-encapsulated sensing components to deliver the components inside an extracellular vesicle (EV) through EV-liposome fusion, allowing for the detection and quantification of target miRNA. For instance, Hong et al. developed a liposome fusion-based CRISPR/Cas13a system by incorporating CRISPR components (Leptotrichia wadei/LwaCas13a, crRNA, and FQ probes) into a liposome to detect miR‐21‐5p present inside the tumor-derived extracellular vesicles of ovarian cancer cell lines. This approach, which required neither RNA nor EV extraction achieved a high degree of specificity in identifying intact EVs containing target miRNA and a liposome fusion efficiency over 80% (Hong et al., 2023).

## 5 Discussion

The high death rate of ovarian cancer patients’ attributes to several factors, including the absence of an early screening technique, the frequency of chemo-resistance, relapse after therapy, bad prognosis and discovery at advanced stage. Although the availability of several biomarkers makes the detection process much easier still, one significant unmet medical need is the identification of more precise molecular biomarkers for the early and specific detection of ovarian cancer. CA-125 which FDA has approved, is still the most effective and prevalent biomarker for ovarian cancer diagnosis in clinics; nevertheless, its low specificity makes it a less reliable approach and obligates the need for more precise biomarkers. In addition to CA-125, other biomarkers have also shown variability in their presence in different physiological conditions in the body, reducing their sensitivity and specificity. This is where miRNA comes into the picture since research has demonstrated its high specificity and sensitivity in detecting ovarian carcinoma. Therefore, to improve the survival rate, the differentiating expression patterns of miRNAs between cancer patients and healthy individuals are increasingly explored as a diagnostic, prognostic and therapeutic target.

Recent research on miRNAs (Tables 2, 3) has established that circulating or tumor miRNAs, either alone or in combination, may be explored as potential novel diagnostic biomarkers for OC, as these significantly contribute to its progression by altering target gene expression. For example, according to Zhang et al., there is a correlation between the FIGO stage of patients and the elevated levels of miR-320a and reduced RASSF8 expression in EOC tissue samples. This suggests that miR-320a plays an oncogenic role in tumour progression via targeting RASSF8, a tumor suppressor gene. In addition to promoting the progression of the tumors, miR-320a was also shown to promote EMT of EOC cells, further contributing towards its oncogenesis (Zhang et al., 2021a). Todeschini and group concluded miR-1246 as a strong diagnostic biomarker for HGSC with a significantly higher expression between HGSOC patients vs. healthy controls and a noticeable diagnostic value having a sensitivity and specificity of 87% and 77% (Todeschini et al., 2017). In addition, Oliveira et al. confirmed an upregulated expression of miR-200c-3p and miR-221-3p in patients with malignant pelvic mass and proved that combining miR-200c-3p and miR-221-3p with CA-125 improved the overall diagnostic accuracy to an interesting level (Oliveira et al., 2019). According to the studies, miRNAs with high sensitivity and specificity, as well as good combined AUC values, such as miR-1246, miR-200c-3p, or a panel of miRNAs including miR-200a, miR-200b, miR-373, and miR-200c may be taken into consideration as a potential diagnostic biomarker for clinical applications. Besides the deregulated levels of miRNAs in OC, several other advantages of miRNAs have also encouraged their potential as biomarkers in OC diagnosis. Such as expression profiling of only a small number of miRNAs can be used to successfully type tissues and determine their malignancy rate (Lu et al., 2005). Further, their small size and resistance to RNAses render them stable at physiological pH and temperature. Another contributing factor towards their stability is their association with argonaut protein or their packaging inside exosomes or vesicles. However, the 3′ and 5′ ends of miRNAs and pre-miRNAs appear to be sensitive to exoribonucleases compared to their precursor pri-miRNAs (Bail et al., 2010). Tumour originated miRNAs can also pass across membranes, tissues, and organs to peripheral blood, adding their value as a potential diagnostic biomarker (Nowak et al., 2015). In addition, miRNAs can be used in therapeutics as an intriguing strategy to target cancer cells effectively because each miRNA typically targets a large number of mRNAs which eventually regulates multiple genes at once.

Despite many pros, employing miRNA expression as a biomarker for disease diagnosis demands certain facts to be considered. For instance, miRNAs in the serum/plasma of ovarian cancer patients differ in histological examinations from miRNAs isolated from surgically removed specimens. Where the former is more significant from the perspective of early diagnosis, the latter is for confirmation of intraoperative diagnosis. Further, to establish miRNA as a clinical biomarker for ovarian cancer, it can be integrated with multiple biomarker panels and could be sensitively detected by employing more recent diagnostic techniques or platforms. Even though methods like meta-analysis and systematic reviews showed great promise for screening dysregulated miRNAs in OC, their strength is still limited because of inter-study heterogeneity and a lack of extractable data limiting the inclusion of studies (Frisk et al., 2023). Another fact that is still at the forefront is that the latest diagnostic methods, such as microfluidics, have made significant developments in recent years and allow real-time monitoring of therapy with good sensitivity and low detection limit; the scalability of these devices has been a critical worry (Moghaddam et al., 2023). As a result, to make the detection process fast and their successful clinical translation, integration of detection techniques and designing more point of care (POC) devices should be a major focus for researchers. Extensive clinical trials and validation studies are also required for such assays.

## 6 Conclusion and future perspective

As a focal point in tumor studies, miRNAs displayed good efficiency as a biological target for disease prognosis, prediction, chemoresistance and chemosensitivity monitoring, early screening, targeted customized therapy, and studying patients’ progression-free survival in ovarian malignancy. Despite significant advancements made in our comprehension of how miRNAs associate with various OC hallmarks and how to apply this understanding to enhance cancer patient diagnosis and prognosis, there are several drawbacks linked to the exploitation of miRNA in diagnostic assay, including variable sample collection and detection approaches, the total cohort included in the study and the diversity among genetic backgrounds. The patient inclusion and exclusion criteria followed by the researchers and heterogeneity in miRNA expression among different samples often make it challenging to identify and validate the miRNAs associated with the disease progression and to develop a miRNA panel with a robust data for such applications. Since miRNAs target both oncogenes and tumor suppressor genes, their role should be looked at carefully while developing a standard panel of miRNAs for diagnosis. Further, disparity among the controls used by the researchers, such as using tissues from benign tumour as controls or using normal ovarian, adds another significant fact to be considered while designing a diagnostic assay as the miRNA expression patterns may entirely differ in different tissues. One of the ways to combat these shortcomings and successfully translate circulating miRNAs as a reliable, non-invasive diagnostic biomarker into extensive clinical settings by including subtype-specific large cohorts of samples to produce more reliable data and prognose patients in a better way. Also, giving more careful consideration to studies specified on understanding the mechanism of miRNA-based OC progression would lead us to its successful application in clinical settings. Analysis of tumour-specific exosomes and other microvesicles linked to OC-related miRNAs may also provide a better understanding of the biological functions of microRNAs. Furthermore, a uniform sample selection and analysis criterion should be standardized and globally adopted to eliminate discrepancies. The most important concern still floating and demands to be resolved when employing miRNAs to improve OC management is developing standardized protocols for sample processing, storage and miRNA isolation & detection and integrating their optimization using various bioinformatic and AI-based algorithms could aid in recruiting miRNA as a possible diagnostic tool for early-stage OC detection. Different diagnostic platforms can be used for fast, reliable and accurate diagnosis of OC, as discussed in the manuscript. Even with ongoing advancements in diagnostic methods, there is still much need for new economically viable commercial gadgets and point-of-care devices for miRNA analysis with proven potential to be translated from laboratory to clinics. Furthermore, in order to thoroughly exploit the usefulness of miRNAs in OC treatment, the potential benefit of combining computational research and clinical trials needs to be emphasized.

## Abbreviations

OC, ovarian cancer; HGSC, high grade serous carcinoma; EOC, epithelial ovarian cancer; ICMR, Indian Council of Medical Research; WHO, World Health Organization; BRCA, breast cancer gene; TVS, transvaginal ultrasonography; CT, computed tomography; CA-125, Carbohydrate antigen; HE-4, human epididymis protein 4; AUC, Area under the ROC Curve; FDA, U.S. Food and Drug Administration; RASSF1A, RAS association domain family protein 1a gene; MLH1, MutL homolog 1; MSH2, MutL homolog 2; AAb, Tumor antigen-associated autoantibodies; PTPRA, Protein Tyrosine Phosphatase Receptor Type A; PTGFR, prostaglandin F receptor; TGF-β, Transforming growth factor-β; UTR, untranslated region; EMT, Epithelial-mesenchymal transition; ZEB1, Zinc Finger E-Box Binding Homeobox 1; ZEB2, Zinc Finger E-Box Binding Homeobox 2; ROS, Reactive oxygen species; FOXL2, Forkhead box protein L2; STK11, Serine/Threonine Kinase 11; AFP, alpha-fetoprotein; LDH, Lactate dehydrogenase.

## References

[B1] Anderson K. S. Cramer D. W. Sibani S. Wallstrom G. Wong J. Park J. (2015). Autoantibody signature for the serologic detection of ovarian cancer. J. proteome Res. 14 (1), 578–586. 10.1021/pr500908n 25365139 PMC4334299

[B2] Asl E. R. Sarabandi S. Shademan B. Dalvandi K. Nourazarian A. (2023). MicroRNA targeting: a novel therapeutic intervention for ovarian cancer. Biochem. Biophysics Rep. 35, 101519. 10.1016/j.bbrep.2023.101519 PMC10382632 37521375

[B3] Atallah G. A. Abd. Aziz N. H. Teik C. K. Shafiee M. N. Kampan N. C. (2021). New predictive biomarkers for ovarian cancer. Diagnostics 11 (3), 465. 10.3390/diagnostics11030465 33800113 PMC7998656

[B4] Austin T. M. (2013). First microRNA mimic enters clinic. Nat. Biotechnol. 31 (7), 577. 10.1038/nbt0713-577 23839128

[B5] Author Anonymous (2023). Cancer stat facts: ovarian cancer. National cancer institute. Available at: https://seer.cancer.gov/statfacts/html/ovary.html (Accessed July 15, 2023).

[B6] Bader A. G. Brown D. Winkler M. (2010). The promise of microRNA replacement therapy. Cancer Res. 70 (18), 7027–7030. 10.1158/0008-5472.CAN-10-2010 20807816 PMC2940943

[B7] Bail S. Swerdel M. Liu H. Jiao X. Goff L. A. Hart R. P. (2010). Differential regulation of microRNA stability. Rna 16 (5), 1032–1039. 10.1261/rna.1851510 20348442 PMC2856875

[B8] Barbu M. G. Condrat C. E. Thompson D. C. Bugnar O. L. Cretoiu D. Toader O. D. (2020). MicroRNA involvement in signaling pathways during viral infection. Front. Cell Dev. Biol. 8, 143. 10.3389/fcell.2020.00143 32211411 PMC7075948

[B9] Bast Jr R. Xu F.-J. Yu Y.-H. Barnhill S. Zhang Z. Mills G. (1998). CA 125: the past and the future. Int. J. Biol. markers 13 (4), 179–187. 10.1177/172460089801300402 10228898

[B10] Bast R. Feeney M. Lazarus H. Nadler L. M. Colvin R. B. Knapp R. C. (1981). Reactivity of a monoclonal antibody with human ovarian carcinoma. J. Clin. investigation 68 (5), 1331–1337. 10.1172/jci110380 PMC370929 7028788

[B11] Beg A. Parveen R. Fouad H. Yahia M. Hassanein A. S. (2022). Role of different non-coding RNAs as ovarian cancer biomarkers. J. Ovarian Res. 15 (1), 72. 10.1186/s13048-022-01002-3 35715825 PMC9206245

[B12] Beg A. Parveen R. Fouad H. Yahia M. Hassanein A. S. (2023). Identification of driver genes and miRNAs in ovarian cancer through an integrated in-silico approach. Biology 12 (2), 192. 10.3390/biology12020192 36829472 PMC9952540

[B13] Bendoraite A. Knouf E. C. Garg K. S. Parkin R. K. Kroh E. M. O'Briant K. C. (2010). Regulation of miR-200 family microRNAs and ZEB transcription factors in ovarian cancer: evidence supporting a mesothelial-to-epithelial transition. Gynecol. Oncol. 116 (1), 117–125. 10.1016/j.ygyno.2009.08.009 19854497 PMC2867670

[B14] Bisht D. Arora A. Sachan M. (2022). Role of DNA De-methylation intermediate ‘5-hydroxymethylcytosine’in ovarian cancer management: a comprehensive review. Biomed. Pharmacother. 155, 113674. 10.1016/j.biopha.2022.113674 36099791

[B15] Boyerinas B. Park S. M. Murmann A. E. Gwin K. Montag A. G. Zillhardt M. (2012). Let‐7 modulates acquired resistance of ovarian cancer to Taxanes via IMP‐1‐mediated stabilization of multidrug resistance 1. Int. J. cancer 130 (8), 1787–1797. 10.1002/ijc.26190 21618519 PMC3230767

[B16] Braicu O.-L. Budisan L. Buiga R. Jurj A. Achimas-Cadariu P. Pop L. A. (2017). miRNA expression profiling in formalin-fixed paraffin-embedded endometriosis and ovarian cancer samples. OncoTargets Ther. 10, 4225–4238. 10.2147/OTT.S137107 PMC5584916 28894379

[B17] Brozovic A. Duran G. E. Wang Y. C. Francisco E. B. Sikic B. I. (2015). The miR-200 family differentially regulates sensitivity to paclitaxel and carboplatin in human ovarian carcinoma OVCAR-3 and MES-OV cells. Mol. Oncol. 9 (8), 1678–1693. 10.1016/j.molonc.2015.04.015 26025631 PMC4788969

[B18] Cai J. Yang C. Yang Q. Ding H. Jia J. Guo J. (2013). Deregulation of let-7e in epithelial ovarian cancer promotes the development of resistance to cisplatin. Oncogenesis 2 (10), e75–e. 10.1038/oncsis.2013.39 24100610 PMC3816216

[B19] Calura E. Fruscio R. Paracchini L. Bignotti E. Ravaggi A. Martini P. (2013). MiRNA landscape in stage I epithelial ovarian cancer defines the histotype specificities. Clin. cancer Res. 19 (15), 4114–4123. 10.1158/1078-0432.CCR-13-0360 23766361

[B20] Cannistra S. A. (2004). Cancer of the ovary. N. Engl. J. Med. 351 (24), 2519–2529. 10.1056/NEJMra041842 15590954

[B21] Cao Q. Lu K. Dai S. Hu Y. Fan W. (2014). Clinicopathological and prognostic implications of the miR-200 family in patients with epithelial ovarian cancer. Int. J. Clin. Exp. Pathology 7 (5), 2392–2401. PMC4069884 24966949

[B22] Cerne K. Hadzialjevic B. Skof E. Verdenik I. Kobal B. (2019). Potential of osteopontin in the management of epithelial ovarian cancer. Radiology Oncol. 53 (1), 105–115. 10.2478/raon-2019-0003 PMC6411016 30712025

[B23] Charkhchi P. Cybulski C. Gronwald J. Wong F. O. Narod S. A. Akbari M. R. (2020). CA125 and ovarian cancer: a comprehensive review. Cancers 12 (12), 3730. 10.3390/cancers12123730 33322519 PMC7763876

[B24] Chen D. Wu Y. Tilley R. D. Gooding J. J. (2022). Rapid and ultrasensitive electrochemical detection of DNA methylation for ovarian cancer diagnosis. Biosens. Bioelectron. 206, 114126. 10.1016/j.bios.2022.114126 35240438

[B25] Chen Y. N. Ren C. C. Yang L. Nai M. M. Xu Y. M. Zhang F. (2019). MicroRNA let-7d-5p rescues ovarian cancer cell apoptosis and restores chemosensitivity by regulating the p53 signaling pathway via HMGA1. Int. J. Oncol. 54 (5), 1771–1784. 10.3892/ijo.2019.4731 30816441

[B26] Chen Z. Xiao Z. Zeng S. Yan Z. (2021). The potential value of microRNA-145 for predicting prognosis in patients with ovarian cancer: a protocol for systematic review and meta-analysis. Medicine 100 (32), e26922. 10.1097/MD.0000000000026922 34397934 PMC8360411

[B27] Cho K. R. Shih I.-M. (2009). Ovarian cancer. Annu. Rev. pathology Mech. Dis. 4, 287–313. 10.1146/annurev.pathol.4.110807.092246 PMC2679364 18842102

[B28] Chugh P. Dittmer D. P. (2012). Potential pitfalls in microRNA profiling. Wiley Interdiscip. Rev. RNA. 3 (5), 601–616. 10.1002/wrna.1120 22566380 PMC3597218

[B29] Cochrane D. R. Howe E. N. Spoelstra N. S. Richer J. K. (2010). Loss of miR-200c: a marker of aggressiveness and chemoresistance in female reproductive cancers. J. Oncol. 2010, 821717. 10.1155/2010/821717 20049172 PMC2798671

[B30] Costa F. P. Batista Junior E. L. Zelmanowicz A. Svedman C. Devenz G. Alves S. (2009). Prostasin, a potential tumor marker in ovarian cancer: a pilot study. Clinics 64, 641–644. 10.1590/S1807-59322009000700006 19606239 PMC2710436

[B31] Cui Y. Hong S. Zhu X. (2020). The accuracy of single MicroRNAs in peripheral blood to diagnose ovarian cancer: an updated meta-analysis. Dis. markers 2020, 1075942. 10.1155/2020/1075942 32025275 PMC6983285

[B32] Dave V. P. Ngo T. A. Pernestig A.-K. Tilevik D. Kant K. Nguyen T. (2019). MicroRNA amplification and detection technologies: opportunities and challenges for point of care diagnostics. Lab. Investig. 99 (4), 452–469. 10.1038/s41374-018-0143-3 30542067

[B33] Davies M. Davey M. G. Miller N. (2022). The potential of MicroRNAs as clinical biomarkers to aid ovarian cancer diagnosis and treatment. Genes 13 (11), 2054. 10.3390/genes13112054 36360295 PMC9690044

[B34] Dochez V. Caillon H. Vaucel E. Dimet J. Winer N. Ducarme G. (2019). Biomarkers and algorithms for diagnosis of ovarian cancer: CA125, HE4, RMI and ROMA, a review. J. ovarian Res. 12, 28–29. 10.1186/s13048-019-0503-7 30917847 PMC6436208

[B35] Ebert M. S. Neilson J. R. Sharp P. A. (2007). MicroRNA sponges: competitive inhibitors of small RNAs in mammalian cells. Nat. methods 4 (9), 721–726. 10.1038/nmeth1079 17694064 PMC3857099

[B37] Ferreira P. Roela R. A. Lopez R. V. M. Estevez-Diz M. D. P. (2020). The prognostic role of microRNA in epithelial ovarian cancer: a systematic review of literature with an overall survival meta-analysis. Oncotarget 11 (12), 1085–1095. 10.18632/oncotarget.27246 32256980 PMC7105164

[B38] Frisk N. L. S. Sørensen A. E. Pedersen O. B. V. Dalgaard L. T. (2023). Circulating microRNAs for early diagnosis of ovarian cancer: a systematic review and meta-analysis. Biomolecules 13 (5), 871. 10.3390/biom13050871 37238740 PMC10216356

[B39] Fujiwara H. Suzuki M. Takeshima N. Takizawa K. Kimura E. Nakanishi T. (2015). Evaluation of human epididymis protein 4 (HE4) and Risk of Ovarian Malignancy Algorithm (ROMA) as diagnostic tools of type I and type II epithelial ovarian cancer in Japanese women. Tumor Biol. 36, 1045–1053. 10.1007/s13277-014-2738-7 PMC4342513 25326813

[B40] Fukagawa S. Miyata K. Yotsumoto F. Kiyoshima C. Nam S. O. Anan H. (2017). MicroRNA‐135a‐3p as a promising biomarker and nucleic acid therapeutic agent for ovarian cancer. Cancer Sci. 108 (5), 886–896. 10.1111/cas.13210 28231414 PMC5448652

[B41] Gandham S. K. Rao M. Shah A. Trivedi M. S. Amiji M. M. (2022). Combination microRNA-based cellular reprogramming with paclitaxel enhances therapeutic efficacy in a relapsed and multidrug-resistant model of epithelial ovarian cancer. Mol. Therapy-Oncolytics 25, 57–68. 10.1016/j.omto.2022.03.005 PMC8971728 35399604

[B42] Gao Y. Meng H. Liu S. Hu J. Zhang Y. Jiao T. (2015). LncRNA-HOST2 regulates cell biological behaviors in epithelial ovarian cancer through a mechanism involving microRNA let-7b. Hum. Mol. Genet. 24 (3), 841–852. 10.1093/hmg/ddu502 25292198

[B43] Gentry-Maharaj A. Blyuss O. Ryan A. Burnell M. Karpinskyj C. Gunu R. (2020). Multi-marker longitudinal algorithms incorporating HE4 and CA125 in ovarian cancer screening of postmenopausal women. Cancers 12 (7), 1931. 10.3390/cancers12071931 32708856 PMC7409061

[B44] Gershenson D. M. (1994). Management of early ovarian cancer: germ cell and sex cord-stromal tumors. Gynecol. Oncol. 55 (3), S562–S572. 10.1006/gyno.1994.1343 7530680

[B45] Ghafoor A. Thomas A. Hassan R. (2018). Targeting mesothelin in ovarian cancer. Oncotarget 9 (90), 36050–36051. 10.18632/oncotarget.26350 30546824 PMC6281414

[B46] Gică N. Peltecu G. Chirculescu R. Gică C. Stoicea M. C. Serbanica A. N. (2022). Ovarian germ cell tumors: pictorial essay. Diagnostics 12 (9), 2050. 10.3390/diagnostics12092050 36140449 PMC9498179

[B47] Goff B. A. (2012). Ovarian cancer: screening and early detection. Obstetrics Gynecol. Clin. 39 (2), 183–194. 10.1016/j.ogc.2012.02.007 22640710

[B48] Gov E. Kori M. Arga K. Y. (2017). Multiomics analysis of tumor microenvironment reveals Gata2 and miRNA-124-3p as potential novel biomarkers in ovarian cancer. Omics a J. Integr. Biol. 21 (10), 603–615. 10.1089/omi.2017.0115 28937943

[B49] Guo J. Yu J. Song X. Mi H. (2017). Serum CA125, CA199 and CEA combined detection for epithelial ovarian cancer diagnosis: a meta-analysis. Open Med. 12 (1), 131–137. 10.1515/med-2017-0020 PMC5471922 28730172

[B50] Guo X. Liu G. Schauer I. G. Yang G. Mercado-Uribe I. Yang F. (2011). Overexpression of the β subunit of human chorionic gonadotropin promotes the transformation of human ovarian epithelial cells and ovarian tumorigenesis. Am. J. pathology 179 (3), 1385–1393. 10.1016/j.ajpath.2011.05.018 PMC3157261 21763678

[B51] Hamidi F. Gilani N. Arabi Belaghi R. Yaghoobi H. Babaei E. Sarbakhsh P. (2023). Identifying potential circulating miRNA biomarkers for the diagnosis and prediction of ovarian cancer using machine-learning approach: application of Boruta. Front. Digital Health 5, 1187578. 10.3389/fdgth.2023.1187578 PMC10445490 37621964

[B52] Hardikar A. A. Farr R. J. Joglekar M. V. (2014). Circulating microRNAs: understanding the limits for quantitative measurement by real‐time PCR. J. Am. Heart Assoc. 3 (1), e000792. 10.1161/JAHA.113.000792 24572259 PMC3959687

[B53] Hasenburg A. Eichkorn D. Vosshagen F. Obermayr E. Geroldinger A. Zeillinger R. (2021). Biomarker-based early detection of epithelial ovarian cancer based on a five-protein signature in patient’s plasma–a prospective trial. BMC cancer 21 (1), 1037–1038. 10.1186/s12885-021-08682-y 34530759 PMC8447799

[B54] Hibbs K. Skubitz K. M. Pambuccian S. E. Casey R. C. Burleson K. M. Oegema Jr T. R. (2004). Differential gene expression in ovarian carcinoma: identification of potential biomarkers. Am. J. pathology 165 (2), 397–414. 10.1016/S0002-9440(10)63306-8 PMC1618570 15277215

[B55] Hilliard T. S. (2018). The impact of mesothelin in the ovarian cancer tumor microenvironment. Cancers 10 (9), 277. 10.3390/cancers10090277 30134520 PMC6162689

[B56] Hong F. Li Y. Xu Y. Zhu L. (2013). Prognostic significance of serum microRNA-221 expression in human epithelial ovarian cancer. J. Int. Med. Res. 41 (1), 64–71. 10.1177/0300060513475759 23569131

[B57] Hong J. S. Son T. Castro C. M. Im H. (2023). CRISPR/Cas13a‐Based MicroRNA detection in tumor‐derived extracellular vesicles. Adv. Sci. 10, 2301766. 10.1002/advs.202301766 PMC10460892 37340600

[B58] Hou J. Y. Chapman J. S. Kalashnikova E. Pierson W. Smith-McCune K. Pineda G. (2022). Circulating tumor DNA monitoring for early recurrence detection in epithelial ovarian cancer. Gynecol. Oncol. 167 (2), 334–341. 10.1016/j.ygyno.2022.09.004 36117009

[B59] Howe E. N. Cochrane D. R. Richer J. K. (2011). Targets of miR-200c mediate suppression of cell motility and anoikis resistance. Breast cancer Res. 13 (2), R45–R15. 10.1186/bcr2867 21501518 PMC3219208

[B60] Hu C. Zhang L. Yang Z. Song Z. Zhang Q. He Y. (2021). Graphene oxide-based qRT-PCR assay enables the sensitive and specific detection of miRNAs for the screening of ovarian cancer. Anal. Chim. Acta. 1174, 338715. 10.1016/j.aca.2021.338715 34247740

[B61] Hu Z.-D. Wei T.-T. Yang M. Ma N. Tang Q.-Q. Qin B.-D. (2015). Diagnostic value of osteopontin in ovarian cancer: a meta-analysis and systematic review. PloS one 10 (5), e0126444. 10.1371/journal.pone.0126444 25951060 PMC4423864

[B62] Huang C-C Sung C-Y Chen Y-S Hsu K-F Lee G-B (2022). “An integrated, multiplex digital PCR-based microfluidic system for quantification of two microrna biomarkers for diagnosis of ovarian cancer,” in 2022 IEEE 35th International Conference on Micro Electro Mechanical Systems Conference (MEMS), Tokyo, Japan, 09-13 January 2022 (IEEE).

[B63] Ibrahim F. F. Jamal R. Syafruddin S. E. Ab Mutalib N. S. Saidin S. MdZin R. R. (2015). MicroRNA-200c and microRNA-31 regulate proliferation, colony formation, migration and invasion in serous ovarian cancer. J. ovarian Res. 8 (1), 56–14. 10.1186/s13048-015-0186-7 26260454 PMC4531514

[B64] Iorio M. V. Visone R. Di Leva G. Donati V. Petrocca F. Casalini P. (2007). MicroRNA signatures in human ovarian cancer. Cancer Res. 67 (18), 8699–8707. 10.1158/0008-5472.CAN-07-1936 17875710

[B65] Islam M. N. Gorgannezhad L. Masud M. K. Tanaka S. Al Hossain M. S. Yamauchi Y. (2018). Graphene oxide-loaded iron oxide superparamagnetic nanoparticles for ultrasensitive electrocatalytic detection of microRNA. ChemElectroChem 5 (17), 2488–2495. 10.1002/celc.201800339

[B66] Islam M. N. Masud M. K. Haque M. H. Hossain M. S. A. Yamauchi Y. Nguyen N. T. (2017). RNA biomarkers: diagnostic and prognostic potentials and recent developments of electrochemical biosensors. Small Methods 1 (7), 1700131. 10.1002/smtd.201700131

[B67] Ismail A. Abulsoud A. I. Fathi D. Elshafei A. El-Mahdy H. A. Elsakka E. G. (2022). The role of miRNAs in ovarian cancer pathogenesis and therapeutic resistance-A focus on signaling pathways interplay. Pathology-Research Pract. 240, 154222. 10.1016/j.prp.2022.154222 36413828

[B68] Ivanov Y. D. Kapustina S. I. Malsagova K. A. Goldaeva K. V. Pleshakova T. O. Galiullin R. A. (2022). “Silicon-On-Insulator”-Based biosensor for the detection of MicroRNA markers of ovarian cancer. Micromachines 14 (1), 70. 10.3390/mi14010070 36677130 PMC9861449

[B69] Ivanova T. I. Klabukov I. D. Krikunova L. I. Poluektova M. V. Sychenkova N. I. Khorokhorina V. A. (2022). Prognostic value of serum transferrin analysis in patients with ovarian cancer and cancer-related functional iron deficiency: a retrospective case–control study. J. Clin. Med. 11 (24), 7377. 10.3390/jcm11247377 36555993 PMC9786287

[B70] Jacobs I. Oram D. Fairbanks J. Turner J. Frost C. Grudzinskas J. (1990). A risk of malignancy index incorporating CA 125, ultrasound and menopausal status for the accurate preoperative diagnosis of ovarian cancer. BJOG Int. J. Obstetrics Gynaecol. 97 (10), 922–929. 10.1111/j.1471-0528.1990.tb02448.x 2223684

[B71] Javdekar R. Maitra N. (2015). Risk of malignancy index (RMI) in evaluation of adnexal mass. J. Obstetrics Gynecol. India 65, 117–121. 10.1007/s13224-014-0609-1 PMC4395584 25883443

[B72] Jordan S. M. Bristow R. E. (2013). Ovarian cancer biomarkers as diagnostic triage tests. Current Biomarker Findings, 35–42.

[B73] Kaijser J. Sayasneh A. Van Hoorde K. Ghaem-Maghami S. Bourne T. Timmerman D. (2014). Presurgical diagnosis of adnexal tumours using mathematical models and scoring systems: a systematic review and meta-analysis. Hum. Reprod. update 20 (3), 449–462. 10.1093/humupd/dmt059 24327552

[B74] Kandettu A. Adiga D. Devi V. Suresh P. S. Chakrabarty S. Radhakrishnan R. (2022). Deregulated miRNA clusters in ovarian cancer: imperative implications in personalized medicine. Genes & Dis. 9 (6), 1443–1465. 10.1016/j.gendis.2021.12.026 PMC9485269 36157483

[B75] Kankanala V. Mukkamalla S. (2023). Carcinoembryonic antigen. Treasure Island (FL): StatPearls Publishing. 35201700

[B76] Kelly P. J. Archbold P. Price J. H. Cardwell C. McCluggage W. G. (2010). Serum CA19. 9 levels are commonly elevated in primary ovarian mucinous tumours but cannot be used to predict the histological subtype. J. Clin. Pathology 63 (2), 169–173. 10.1136/jcp.2009.072355 20154039

[B77] Kilic T. Erdem A. Ozsoz M. Carrara S. (2018). microRNA biosensors: opportunities and challenges among conventional and commercially available techniques. Biosens. Bioelectron. 99, 525–546. 10.1016/j.bios.2017.08.007 28823978

[B78] Kim V. N. (2005). MicroRNA biogenesis: coordinated cropping and dicing. Nat. Rev. Mol. Cell Biol. 6 (5), 376–385. 10.1038/nrm1644 15852042

[B79] Kluiver J. Slezak-Prochazka I. Smigielska-Czepiel K. Halsema N. Kroesen B.-J. van den Berg A. (2012). Generation of miRNA sponge constructs. Methods. 58 (2), 113–117. 10.1016/j.ymeth.2012.07.019 22836127

[B80] Klymenko Y. Bos B. Campbell L. Loughran E. Liu Y. Yang J. (2020). Lysophosphatidic acid modulates ovarian cancer multicellular aggregate assembly and metastatic dissemination. Sci. Rep. 10 (1), 10877. 10.1038/s41598-020-67565-7 32616784 PMC7331713

[B81] Kobayashi H. Suzuki M. Hirashima Y. Terao T. (2003). The protease inhibitor bikunin, a novel anti-metastatic agent. 10.1515/BC.2003.083 12817471

[B82] Koscianska E. Starega-Roslan J. Sznajder L. J. Olejniczak M. Galka-Marciniak P. Krzyzosiak W. J. (2011). Northern blotting analysis of microRNAs, their precursors and RNA interference triggers. BMC Mol. Biol. 12, 14–17. 10.1186/1471-2199-12-14 21481235 PMC3080303

[B83] Koshiol J. Wang E. Zhao Y. Marincola F. Landi M. T. (2010). Strengths and limitations of laboratory procedures for microRNA detection. Cancer Epidemiol. biomarkers Prev. 19 (4), 907–911. 10.1158/1055-9965.EPI-10-0071 20332265 PMC2852469

[B84] Koshiyama M. Matsumura N. Konishi I. (2014). Recent concepts of ovarian carcinogenesis: type I and type II. BioMed Res. Int. 2014, 934261. 10.1155/2014/934261 24868556 PMC4017729

[B85] Krasniqi E. Sacconi A. Marinelli D. Pizzuti L. Mazzotta M. Sergi D. (2021). MicroRNA-based signatures impacting clinical course and biology of ovarian cancer: a miRNOmics study. Biomark. Res. 9 (1), 57–17. 10.1186/s40364-021-00289-6 34256855 PMC8276429

[B86] Kristjansdottir B. Levan K. Partheen K. Sundfeldt K. (2013). Diagnostic performance of the biomarkers HE4 and CA125 in type I and type II epithelial ovarian cancer. Gynecol. Oncol. 131 (1), 52–58. 10.1016/j.ygyno.2013.07.094 23891789

[B87] Kuang Y. Xu H. Lu F. Meng J. Yi Y. Yang H. (2021). Inhibition of microRNA let‐7b expression by KDM2B promotes cancer progression by targeting EZH2 in ovarian cancer. Cancer Sci. 112 (1), 231–242. 10.1111/cas.14708 33091189 PMC7780014

[B88] Langhe R. Norris L. Saadeh F. A. Blackshields G. Varley R. Harrison A. (2015). A novel serum microRNA panel to discriminate benign from malignant ovarian disease. Cancer Lett. 356 (2), 628–636. 10.1016/j.canlet.2014.10.010 25451316

[B89] Lee T. Teng T. Z. J. Shelat V. G. (2020). Carbohydrate antigen 19-9—tumor marker: past, present, and future. World J. Gastrointest. Surg. 12 (12), 468–490. 10.4240/wjgs.v12.i12.468 33437400 PMC7769746

[B90] Leon S. Shapiro B. Sklaroff D. Yaros M. (1977). Free DNA in the serum of cancer patients and the effect of therapy. Cancer Res. 37 (3), 646–650. 837366

[B91] Lertkhachonsuk A. A. Buranawongtrakoon S. Lekskul N. Rermluk N. Wee-Stekly W. W. Charakorn C. (2020). Serum CA19-9, CA-125 and CEA as tumor markers for mucinous ovarian tumors. J. Obstetrics Gynaecol. Res. 46 (11), 2287–2291. 10.1111/jog.14427 PMC7693209 32830422

[B92] Leskelä S. Leandro-García L. J. Mendiola M. Barriuso J. Inglada-Pérez L. Muñoz I. (2011). The miR-200 family controls beta-tubulin III expression and is associated with paclitaxel-based treatment response and progression-free survival in ovarian cancer patients. Endocrine-related cancer 18 (1), 85–95. 10.1677/ERC-10-0148 21051560

[B93] Li Y.-Y. Zhang W.-C. Zhang J.-L. Zheng C.-J. Zhu H. Yu H.-M. (2015). Plasma levels of lysophosphatidic acid in ovarian cancer versus controls: a meta-analysis. Lipids Health Dis. 14, 72–79. 10.1186/s12944-015-0071-9 26174150 PMC4501043

[B94] Lin Y.-H. Wu C.-H. Fu H.-C. Chen Y.-J. Chen Y.-Y. Ou Y.-C. (2020). Prognostic significance of elevated pretreatment serum levels of CEA and CA-125 in epithelial ovarian cancer. Cancer Biomarkers 28 (3), 285–292. 10.3233/CBM-201455 32390605 PMC12662360

[B95] Liu C.-G. Calin G. A. Volinia S. Croce C. M. (2008). MicroRNA expression profiling using microarrays. Nat. Protoc. 3 (4), 563–578. 10.1038/nprot.2008.14 18388938

[B96] Low J. J. Ilancheran A. Ng J. S. (2012). Malignant ovarian germ-cell tumours. Best Pract. Res. Clin. obstetrics Gynaecol. 26 (3), 347–355. 10.1016/j.bpobgyn.2012.01.002 22301054

[B97] Lu J. Getz G. Miska E. A. Alvarez-Saavedra E. Lamb J. Peck D. (2005). MicroRNA expression profiles classify human cancers. nature 435 (7043), 834–838. 10.1038/nature03702 15944708

[B98] Lu L. Katsaros D. Rigault de la Longrais I. A. Sochirca O. Yu H. (2007). Hypermethylation of let-7a-3 in epithelial ovarian cancer is associated with low insulin-like growth factor-II expression and favorable prognosis. Cancer Res. 67 (21), 10117–10122. 10.1158/0008-5472.CAN-07-2544 17974952

[B99] Lu L. Schwartz P. Scarampi L. Rutherford T. Canuto E. M. Yu H. (2011). MicroRNA let-7a: a potential marker for selection of paclitaxel in ovarian cancer management. Gynecol. Oncol. 122 (2), 366–371. 10.1016/j.ygyno.2011.04.033 21571355

[B100] Luo T. Jiang Y. Yang J. (2021). Long noncoding RNA LINC01554 as a novel biomarker for diagnosis and prognosis prediction of epithelial ovarian cancer. Dis. Markers 2021, 1244612. 10.1155/2021/1244612 34422133 PMC8371612

[B101] Ma J. Zhan Y. Xu Z. Li Y. Luo A. Ding F. (2017). ZEB1 induced miR-99b/let-7e/miR-125a cluster promotes invasion and metastasis in esophageal squamous cell carcinoma. Cancer Lett. 398, 37–45. 10.1016/j.canlet.2017.04.006 28408353

[B102] Macuks R. Baidekalna I. Gritcina J. Avdejeva A. Donina S. (2010). Apolipoprotein A1 and transferrin as biomarkers in ovarian cancer diagnostics. Acta Chir. Latv. 10 (2), 16–20. 10.2478/v10163-011-0003-3

[B103] Mateescu B. Batista L. Cardon M. Gruosso T. De Feraudy Y. Mariani O. (2011). miR-141 and miR-200a act on ovarian tumorigenesis by controlling oxidative stress response. Nat. Med. 17 (12), 1627–1635. 10.1038/nm.2512 22101765

[B104] Matsuzaki H. Kobayashi H. Yagyu T. Wakahara K. Kondo T. Kurita N. (2005). Plasma bikunin as a favorable prognostic factor in ovarian cancer. J. Clin. Oncol. 23 (7), 1463–1472. 10.1200/JCO.2005.03.010 15735122

[B105] Mayer T. Oesinghaus L. Simmel F. C. (2022). Toehold-mediated strand displacement in random sequence pools. J. Am. Chem. Soc. 145 (1), 634–644. 10.1021/jacs.2c11208 36571481

[B106] Meden H. Fattahi-Meibodi A. (1998). CA 125 in benign gynecological conditions. Int. J. Biol. markers 13 (4), 231–237. 10.1177/172460089801300411 10228907

[B107] Meng X. Müller V. Milde-Langosch K. Trillsch F. Pantel K. Schwarzenbach H. (2016). Diagnostic and prognostic relevance of circulating exosomal miR-373, miR-200a, miR-200b and miR-200c in patients with epithelial ovarian cancer. Oncotarget 7 (13), 16923–16935. 10.18632/oncotarget.7850 26943577 PMC4941360

[B108] Mhatre A. Koroth J. Manjunath M. Kumar S. S. Gawari R. Choudhary B. (2023). Multi-omics analysis of the Indian ovarian cancer cohort revealed histotype-specific mutation and gene expression patterns. Front. Genet. 14, 1102114. 10.3389/fgene.2023.1102114 37091785 PMC10117685

[B109] Moazampour M. Zare H. R. Shekari Z. (2021). Femtomolar determination of an ovarian cancer biomarker (miR-200a) in blood plasma using a label free electrochemical biosensor based on L-cysteine functionalized ZnS quantum dots. Anal. Methods 13 (17), 2021–2029. 10.1039/d1ay00330e 33956002

[B110] Moghaddam F. D. Dadgar D. Esmaeili Y. Babolmorad S. Ilkhani E. Rafiee M. (2023). Microfluidic platforms in diagnostic of ovarian cancer. Environ. Res. 237, 117084. 10.1016/j.envres.2023.117084 37683792

[B111] Mohammed B. T. Mustafa S. I. Zeebaree B. K. (2023). Identification of ovarian cancer using *in silico*-Based analysis of the downregulated expressed miRNAs. Egypt. Acad. J. Biol. Sci. C, Physiology Mol. Biol. 15 (2), 309–323. 10.21608/eajbsc.2023.317702

[B112] Mok S. C. Chao J. Skates S. Wong K.-K. Yiu G. K. Muto M. G. (2001). Prostasin, a potential serum marker for ovarian cancer: identification through microarray technology. J. Natl. Cancer Inst. 93 (19), 1458–1464. 10.1093/jnci/93.19.1458 11584061

[B113] Mollasalehi H. Shajari E. (2021). A colorimetric nano-biosensor for simultaneous detection of prevalent cancers using unamplified cell-free ribonucleic acid biomarkers. Bioorg. Chem. 107, 104605. 10.1016/j.bioorg.2020.104605 33421955

[B114] Monroig P. C. Chen L. Zhang S. Calin G. A. (2015). Small molecule compounds targeting miRNAs for cancer therapy. Adv. drug Deliv. Rev. 81, 104–116. 10.1016/j.addr.2014.09.002 25239236 PMC4461213

[B115] Moore R. G. McMeekin D. S. Brown A. K. DiSilvestro P. Miller M. C. Allard W. J. (2009). A novel multiple marker bioassay utilizing HE4 and CA125 for the prediction of ovarian cancer in patients with a pelvic mass. Gynecol. Oncol. 112 (1), 40–46. 10.1016/j.ygyno.2008.08.031 18851871 PMC3594094

[B116] Muller C. Y. (2010). Doctor, should I get this new ovarian cancer test—ova1? LWW, 246–247. 10.1097/AOG.0b013e3181e934ba 20664381

[B118] Murph M. M. Liu W. Yu S. Lu Y. Hall H. Hennessy B. T. (2009). Lysophosphatidic acid-induced transcriptional profile represents serous epithelial ovarian carcinoma and worsened prognosis. PloS one 4 (5), e5583. 10.1371/journal.pone.0005583 19440550 PMC2679144

[B119] Nahm F. S. (2022). Receiver operating characteristic curve: overview and practical use for clinicians. Korean J. Anesthesiol. 75 (1), 25–36. 10.4097/kja.21209 35124947 PMC8831439

[B120] Nakano H. Yamada Y. Miyazawa T. Yoshida T. (2013). Gain-of-function microRNA screens identify miR-193a regulating proliferation and apoptosis in epithelial ovarian cancer cells. Int. J. Oncol. 42 (6), 1875–1882. 10.3892/ijo.2013.1896 23588298 PMC3699598

[B121] Naumann R. W. Brown J. (2018). Ovarian cancer screening with the risk of ovarian cancer algorithm (ROCA): good, bad, or just expensive? Gynecol. Oncol. 149 (1), 117–120. 10.1016/j.ygyno.2018.01.029 29398069

[B122] Navyatha B. Nara S. (2019). Theranostic nanostructures for ovarian cancer. Crit. Reviews™ Ther. Drug Carr. Syst. 36 (4), 305–371. 10.1615/CritRevTherDrugCarrierSyst.2018025589 31679190

[B123] Nowak M. Janas Ł. Stachowiak G. Stetkiewicz T. Wilczyński J. R. (2015). Current clinical application of serum biomarkers to detect ovarian cancer. Menopause Review/Przegląd Menopauzalny 14 (4), 254–259. 10.5114/pm.2015.55887 PMC4733894 26848298

[B124] Obata K. Morland S. J. Watson R. H. Hitchcock A. Chenevix-Trench G. Thomas E. J. (1998). Frequent PTEN/MMAC mutations in endometrioid but not serous or mucinous epithelial ovarian tumors. Cancer Res. 58 (10), 2095–2097. 9605750

[B125] Oliveira D. N. P. Carlsen A. L. Heegaard N. H. Prahm K. P. Christensen I. J. Høgdall C. K. (2019). Diagnostic plasma miRNA-profiles for ovarian cancer in patients with pelvic mass. PLoS One 14 (11), e0225249. 10.1371/journal.pone.0225249 31738788 PMC6860451

[B126] Ouyang T. Liu Z. Han Z. Ge Q. (2019). MicroRNA detection specificity: recent advances and future perspective. Anal. Chem. 91 (5), 3179–3186. 10.1021/acs.analchem.8b05909 30702270

[B127] Pal M. K. Jaiswar S. P. Dwivedi V. N. Tripathi A. K. Dwivedi A. Sankhwar P. (2015). MicroRNA: a new and promising potential biomarker for diagnosis and prognosis of ovarian cancer. Cancer Biol. Med. 12 (4), 328–341. 10.7497/j.issn.2095-3941.2015.0024 26779370 PMC4706521

[B128] Palacios J. Gamallo C. (1998). Mutations in the beta-catenin gene (CTNNB1) in endometrioid ovarian carcinomas. Cancer Res. 58 (7), 1344–1347. 9537226

[B129] Panda H. Pelakh L. Chuang T.-D. Luo X. Bukulmez O. Chegini N. (2012). Endometrial miR-200c is altered during transformation into cancerous states and targets the expression of ZEBs, VEGFA, FLT1, IKKβ, KLF9, and FBLN5. Reprod. Sci. 19 (8), 786–796. 10.1177/1933719112438448 22569286 PMC4046309

[B130] Park S.-M. Gaur A. B. Lengyel E. Peter M. E. (2008). The miR-200 family determines the epithelial phenotype of cancer cells by targeting the E-cadherin repressors ZEB1 and ZEB2. Genes & Dev. 22 (7), 894–907. 10.1101/gad.1640608 18381893 PMC2279201

[B131] Pecot C. V. Rupaimoole R. Yang D. Akbani R. Ivan C. Lu C. (2013). Tumour angiogenesis regulation by the miR-200 family. Nat. Commun. 4 (1), 2427. 10.1038/ncomms3427 24018975 PMC3904438

[B132] Prahm K. P. Høgdall C. K. Karlsen M. A. Christensen I. J. Novotny G. W. Høgdall E. (2021). MicroRNA characteristics in epithelial ovarian cancer. Plos one 16 (6), e0252401. 10.1371/journal.pone.0252401 34086724 PMC8177468

[B133] Prislei S. Martinelli E. Mariani M. Raspaglio G. Sieber S. Ferrandina G. (2013). MiR-200c and HuR in ovarian cancer. BMC cancer 13, 72–14. 10.1186/1471-2407-13-72 23394580 PMC3576328

[B134] Qiu Y. Chen Y. Agbede O. Eshaghi E. Peng C. (2022). Circular RNAs in epithelial ovarian cancer: from biomarkers to therapeutic targets. Cancers 14 (22), 5711. 10.3390/cancers14225711 36428803 PMC9688053

[B135] Rastogi M. Gupta S. Sachan M. (2016). Biomarkers towards ovarian cancer diagnostics: present and future prospects. Braz. archives Biol. Technol. 59. 10.1590/1678-4324-2016160070

[B136] Schmittgen T. D. Jiang J. Liu Q. Yang L. (2004). A high‐throughput method to monitor the expression of microRNA precursors. Nucleic acids Res. 32 (4), e43–e. 10.1093/nar/gnh040 14985473 PMC390315

[B137] Schultz K. A. P. Harris A. K. Schneider D. T. Young R. H. Brown J. Gershenson D. M. (2016). Ovarian sex cord-stromal tumors. J. Oncol. Pract. 12 (10), 940–946. 10.1200/JOP.2016.016261 27858560 PMC5063189

[B138] Shan S. J. Scorilas A. Katsaros D. Rigault de la Longrais I. Massobrio M. Diamandis E. P. (2006). Unfavorable prognostic value of human kallikrein 7 quantified by ELISA in ovarian cancer cytosols. Clin. Chem. 52 (10), 1879–1886. 10.1373/clinchem.2006.071456 16916986

[B139] Shen G. Ghazizadeh M. Kawanami O. Shimizu H. Jin E. Araki T. (2000). Prognostic significance of vascular endothelial growth factor expression in human ovarian carcinoma. Br. J. cancer 83 (2), 196–203. 10.1054/bjoc.2000.1228 10901370 PMC2363477

[B140] Shi C. Zhang Z. (2016). The prognostic value of the miR‐200 family in ovarian cancer: a meta‐analysis. Acta Obstetricia Gynecol. Scand. 95 (5), 505–512. 10.1111/aogs.12883 26910180

[B141] Shi H. Zhou Y. Jia E. Pan M. Bai Y. Ge Q. (2021). Bias in RNA-seq library preparation: current challenges and solutions. BioMed Res. Int. 2021, 6647597. 10.1155/2021/6647597 33987443 PMC8079181

[B142] Shi L. Zhang S. Wu H. Zhang L. Dai X. Hu J. (2013). MiR-200c increases the radiosensitivity of non-small-cell lung cancer cell line A549 by targeting VEGF-VEGFR2 pathway. PloS one 8 (10), e78344. 10.1371/journal.pone.0078344 24205206 PMC3813610

[B143] Shi M. Mu Y. Zhang H. Liu M. Wan J. Qin X. (2018). MicroRNA-200 and microRNA-30 family as prognostic molecular signatures in ovarian cancer: a meta-analysis. Medicine 97 (32), e11505. 10.1097/MD.0000000000011505 30095616 PMC6133642

[B144] Skates S. J. (2012). Ovarian cancer screening: development of the risk of ovarian cancer algorithm (ROCA) and ROCA screening trials. Int. J. Gynecol. Cancer 22 (S1), S24–S26. 10.1097/IGC.0b013e318256488a 22543916 PMC3572791

[B145] Song K. Artibani M. (2023). The role of DNA methylation in ovarian cancer chemoresistance: a narrative review. Health Sci. Rep. 6 (5), e1235. 10.1002/hsr2.1235 37123549 PMC10140645

[B146] Staicu C. E. Predescu D.-V. Rusu C. M. Radu B. M. Cretoiu D. Suciu N. (2020). Role of microRNAs as clinical cancer biomarkers for ovarian cancer: a short overview. Cells 9 (1), 169. 10.3390/cells9010169 31936634 PMC7016727

[B147] Stewart C. Ralyea C. Lockwood S. (2019). Ovarian cancer: an integrated review. Seminars in oncology nursing. Elsevier. 10.1016/j.soncn.2019.02.001 30867104

[B148] Sun G. Chen C. Li X. Hong S. Gu C. Shi X. (2023). Rapid microRNA detection method based on DNA strand displacement for ovarian cancer cells. J. Cancer 14 (5), 707–716. 10.7150/jca.81050 37056384 PMC10088887

[B149] Sun N. Zhang Q. Xu C. Zhao Q. Ma Y. Lu X. (2014). Molecular regulation of ovarian cancer cell invasion. Tumor Biol. 35, 11359–11366. 10.1007/s13277-014-2434-7 25119590

[B150] Sung C.-Y. Huang C.-C. Chen Y.-S. Lee G.-B. (2021). “Extraction and quantification of microrna biomarkers for diagnosis of ovarian cancer on an integrated microfluidic platform,” in 2021 IEEE 34th International Conference on Micro Electro Mechanical Systems (MEMS), Gainesville, FL, USA, 25-29 January 2021 (IEEE).

[B151] Suryawanshi S. Vlad A. M. Lin H.-M. Mantia-Smaldone G. Laskey R. Lee M. (2013). Plasma microRNAs as novel biomarkers for endometriosis and endometriosis-associated ovarian cancer. Clin. Cancer Res. 19 (5), 1213–1224. 10.1158/1078-0432.CCR-12-2726 23362326 PMC3596045

[B152] Tamakoshi K. Kikkawa F. Shibata K. Tomoda K. Obata N. H. Wakahara F. (1996). Clinical value of CA125, CA19-9, CEA, CA72-4, and TPA in borderline ovarian tumor. Gynecol. Oncol. 62 (1), 67–72. 10.1006/gyno.1996.0191 8690294

[B153] Tamir A. Jag U. Sarojini S. Schindewolf C. Tanaka T. Gharbaran R. (2014). Kallikrein family proteases KLK6 and KLK7 are potential early detection and diagnostic biomarkers for serous and papillary serous ovarian cancer subtypes. J. ovarian Res. 7 (1), 109–115. 10.1186/s13048-014-0109-z 25477184 PMC4271347

[B154] Tanaka Y. Kobayashi H. Suzuki M. Kanayama N. Suzuki M. Terao T. (2004). Upregulation of bikunin in tumor-infiltrating macrophages as a factor of favorable prognosis in ovarian cancer. Gynecol. Oncol. 94 (3), 725–734. 10.1016/j.ygyno.2004.06.012 15350365

[B155] Tang Z. Ow G. S. Thiery J. P. Ivshina A. V. Kuznetsov V. A. (2014). Meta‐analysis of transcriptome reveals let-7b as an unfavorable prognostic biomarker and predicts molecular and clinical subclasses in high-grade serous ovarian carcinoma. Int. J. cancer 134 (2), 306–318. 10.1002/ijc.28371 23825028

[B156] Taylor S. C. Nadeau K. Abbasi M. Lachance C. Nguyen M. Fenrich J. (2019). The ultimate qPCR experiment: producing publication quality, reproducible data the first time. Trends Biotechnol. 37 (7), 761–774. 10.1016/j.tibtech.2018.12.002 30654913

[B157] Teng Y. Su X. Zhang X. Zhang Y. Li C. Niu W. (2016). miRNA-200a/c as potential biomarker in epithelial ovarian cancer (EOC): evidence based on miRNA meta-signature and clinical investigations. Oncotarget 7 (49), 81621–81633. 10.18632/oncotarget.13154 27835595 PMC5348417

[B158] Timmerman D. Valentin L. Bourne T. Collins W. Verrelst H. Vergote I. (2000). Terms, definitions and measurements to describe the sonographic features of adnexal tumors: a consensus opinion from the International Ovarian Tumor Analysis (IOTA) Group. Official J. Int. Soc. Ultrasound Obstetrics Gynecol. 16 (5), 500–505. 10.1046/j.1469-0705.2000.00287.x 11169340

[B159] Tingulstad S. Hagen B. Skjeldestad F. E. Halvorsen T. Nustad K. Onsrud M. (1999). The risk-of-malignancy index to evaluate potential ovarian cancers in local hospitals. Obstetrics Gynecol. 93 (3), 448–452. 10.1097/00006250-199903000-00028 10074998

[B160] Todeschini P. Salviato E. Paracchini L. Ferracin M. Petrillo M. Zanotti L. (2017). Circulating miRNA landscape identifies miR-1246 as promising diagnostic biomarker in high-grade serous ovarian carcinoma: a validation across two independent cohorts. Cancer Lett. 388, 320–327. 10.1016/j.canlet.2016.12.017 28017893

[B161] Tripathi P. Kumar A. Sachan M. Gupta S. Nara S. (2020b). Aptamer-gold nanozyme based competitive lateral flow assay for rapid detection of CA125 in human serum. Biosens. Bioelectron. 165, 112368. 10.1016/j.bios.2020.112368 32729500

[B162] Tripathi P. Sachan M. Nara S. (2020a). Novel ssDNA ligand against ovarian cancer biomarker CA125 with promising diagnostic potential. Front. Chem. 8, 400. 10.3389/fchem.2020.00400 32500059 PMC7242751

[B163] Ueland F. R. (2017). A perspective on ovarian cancer biomarkers: past, present and yet-to-come. Diagnostics 7 (1), 14. 10.3390/diagnostics7010014 28282875 PMC5373023

[B164] Vandghanooni S. Sanaat Z. Barar J. Adibkia K. Eskandani M. Omidi Y. (2021). Recent advances in aptamer-based nanosystems and microfluidics devices for the detection of ovarian cancer biomarkers. TrAC Trends Anal. Chem. 143, 116343. 10.1016/j.trac.2021.116343

[B165] Vang S. Wu H.-T. Fischer A. Miller D. H. MacLaughlan S. Douglass E. (2013). Identification of ovarian cancer metastatic miRNAs. PloS one 8 (3), e58226. 10.1371/journal.pone.0058226 23554878 PMC3595263

[B166] Vernon M. Lambert B. Meryet-Figuière M. Brotin E. Weiswald L.-B. Paysant H. (2020). Functional miRNA screening identifies wide-ranging antitumor properties of miR-3622b-5p and reveals a new therapeutic combination strategy in ovarian tumor organoids. Mol. Cancer Ther. 19 (7), 1506–1519. 10.1158/1535-7163.MCT-19-0510 32371581

[B167] Wan Q. Liu Y. Lv B. Chen X. (2021). Correlation of molecular tumor markers CA125, HE4, and CEA with the development and progression of epithelial ovarian cancer. Iran. J. Public Health 50 (6), 1197–1205. 10.18502/ijph.v50i6.6418 34540740 PMC8410970

[B168] Wang H. Wang T. Shi W. Liu Y. Chen L. Li Z. (2017). Comprehensive analysis on diagnostic value of circulating miRNAs for patients with ovarian cancer. Oncotarget 8 (39), 66620–66628. 10.18632/oncotarget.18129 29029542 PMC5630442

[B169] Wang L. Mezencev R. Švajdler M. Benigno B. B. McDonald J. F. (2014b). Ectopic over-expression of miR-429 induces mesenchymal-to-epithelial transition (MET) and increased drug sensitivity in metastasizing ovarian cancer cells. Gynecol. Oncol. 134 (1), 96–103. 10.1016/j.ygyno.2014.04.055 24802724

[B170] Wang L. Zhu M.-J. Ren A.-M. Wu H.-F. Han W.-M. Tan R.-Y. (2014a). A ten-microRNA signature identified from a genome-wide microRNA expression profiling in human epithelial ovarian cancer. PloS one 9 (5), e96472. 10.1371/journal.pone.0096472 24816756 PMC4015980

[B171] Wang W. Yin Y. Shan X. Zhou X. Liu P. Cao Q. (2019a). The value of plasma-based microRNAs as diagnostic biomarkers for ovarian cancer. Am. J. Med. Sci. 358 (4), 256–267. 10.1016/j.amjms.2019.07.005 31353030

[B172] Wang X. Kong D. Wang C. Ding X. Zhang L. Zhao M. (2019b). Circulating microRNAs as novel potential diagnostic biomarkers for ovarian cancer: a systematic review and updated meta-analysis. J. Ovarian Res. 12, 24–12. 10.1186/s13048-019-0482-8 30898156 PMC6427862

[B173] Wang Y. Bao W. Liu Y. Wang S. Xu S. Li X. (2018a). miR-98-5p contributes to cisplatin resistance in epithelial ovarian cancer by suppressing miR-152 biogenesis via targeting Dicer1. Cell death Dis. 9 (5), 447. 10.1038/s41419-018-0390-7 29670086 PMC5906447

[B174] Wang Y. Kim S. Kim I.-M. (2014c). Regulation of metastasis by microRNAs in ovarian cancer. Front. Oncol. 4, 143. 10.3389/fonc.2014.00143 24959422 PMC4050529

[B175] Wang Z. Ji G. Wu Q. Feng S. Zhao Y. Cao Z. (2018b). Integrated microarray meta-analysis identifies miRNA-27a as an oncogene in ovarian cancer by inhibiting FOXO1. Life Sci. 210, 263–270. 10.1016/j.lfs.2018.08.043 30138596

[B176] Webb P. M. Jordan S. J. (2017). Epidemiology of epithelial ovarian cancer. Best Pract. Res. Clin. obstetrics Gynaecol. 41, 3–14. 10.1016/j.bpobgyn.2016.08.006 27743768

[B177] Wei S. Wang Y. Xu H. Kuang Y. (2015). Screening of potential biomarkers for chemoresistant ovarian carcinoma with miRNA expression profiling data by bioinformatics approach. Oncol. Lett. 10 (4), 2427–2431. 10.3892/ol.2015.3610 26622864 PMC4580032

[B178] White N. Chow T.-F. Mejia-Guerrero S. Diamandis M. Rofael Y. Faragalla H. (2010). Three dysregulated miRNAs control kallikrein 10 expression and cell proliferation in ovarian cancer. Br. J. cancer 102 (8), 1244–1253. 10.1038/sj.bjc.6605634 20354523 PMC2856011

[B179] World Health Organization classification (2023). PathologyOutlines.com. Available at: https://www.pathologyoutlines.com/topic/ovarytumorwhoclassif.html (Accessed July 15, 2023).

[B180] Wu L. Shang W. Zhao H. Rong G. Zhang Y. Xu T. (2019). *In silico* screening of circulating microRNAs as potential biomarkers for the diagnosis of ovarian cancer. Dis. markers 2019, 7541857. 10.1155/2019/7541857 31467618 PMC6701281

[B181] Xia J. Li S. Liu S. Zhang L. (2023). Aldehyde dehydrogenase in solid tumors and other diseases: potential biomarkers and therapeutic targets. MedComm 4 (1), e195. 10.1002/mco2.195 36694633 PMC9842923

[B182] Xiao M. Cai J. Cai L. Jia J. Xie L. Zhu Y. (2017). Let-7e sensitizes epithelial ovarian cancer to cisplatin through repressing DNA double strand break repair. J. ovarian Res. 10 (1), 24–13. 10.1186/s13048-017-0321-8 28376831 PMC5379542

[B183] Xu Y.-Z. Xi Q.-H. Ge W.-L. Zhang X.-Q. (2013). Identification of serum microRNA-21 as a biomarker for early detection and prognosis in human epithelial ovarian cancer. Asian Pac. J. Cancer Prev. 14 (2), 1057–1060. 10.7314/apjcp.2013.14.2.1057 23621186

[B184] Yaghoobi H. Babaei E. Hussen B. M. Emami A. (2020). EBST: an evolutionary multi-objective optimization based tool for discovering potential biomarkers in ovarian cancer. IEEE/ACM Trans. Comput. Biol. Bioinforma. 18 (6), 2384–2393. 10.1109/TCBB.2020.2993150 32396098

[B185] Yang A. Wang X. Yu C. Jin Z. Wei L. Cao J. (2017). microRNA-494 is a potential prognostic marker and inhibits cellular proliferation, migration and invasion by targeting SIRT1 in epithelial ovarian cancer. Oncol. Lett. 14 (3), 3177–3184. 10.3892/ol.2017.6501 28927063 PMC5588040

[B186] Yang F. Tang J. Zhao Z. Zhao C. Xiang Y. (2021). Circulating tumor DNA: a noninvasive biomarker for tracking ovarian cancer. Reproductive Biol. Endocrinol. 19, 178–212. 10.1186/s12958-021-00860-8 PMC8641226 34861867

[B187] Yang N. Kaur S. Volinia S. Greshock J. Lassus H. Hasegawa K. (2008). MicroRNA microarray identifies Let-7i as a novel biomarker and therapeutic target in human epithelial ovarian cancer. Cancer Res. 68 (24), 10307–10314. 10.1158/0008-5472.CAN-08-1954 19074899 PMC2762326

[B188] Yemelyanova A. Cosin J. Bidus M. Boice C. Seidman J. (2008). Pathology of stage I versus stage III ovarian carcinoma with implications for pathogenesis and screening. Int. J. Gynecol. Cancer 18 (3), 465–469. 10.1111/j.1525-1438.2007.01058.x 17868343

[B189] Yokoi A. Matsuzaki J. Yamamoto Y. Yoneoka Y. Takahashi K. Shimizu H. (2018). Integrated extracellular microRNA profiling for ovarian cancer screening. Nat. Commun. 9 (1), 4319. 10.1038/s41467-018-06434-4 30333487 PMC6192980

[B190] Záveský L. Jandáková E. Weinberger V. Minář L. Hanzikova V. Dušková D. (2019). Ovarian cancer: differentially expressed microRNAs in tumor tissue and cell-free ascitic fluid as potential novel biomarkers. Cancer Investig. 37 (9), 440–452. 10.1080/07357907.2019.1663208 31530033

[B191] Zhan L. Li J. Wei B. (2018). Long non-coding RNAs in ovarian cancer. J. Exp. Clin. Cancer Res. 37 (1), 120–213. 10.1186/s13046-018-0793-4 29921308 PMC6008930

[B192] Zhang B. Tian L. Xie J. Chen G. Wang F. (2020). Targeting miRNAs by natural products: a new way for cancer therapy. Biomed. Pharmacother. 130, 110546. 10.1016/j.biopha.2020.110546 32721631

[B193] Zhang L. Chen H. He F. Zhang S. Li A. Zhang A. (2021a). MicroRNA-320a promotes epithelial ovarian cancer cell proliferation and invasion by targeting RASSF8. Front. Oncol. 11, 581932. 10.3389/fonc.2021.581932 33718138 PMC7947674

[B194] Zhang L. Hu C. Huang Z. Li Z. Zhang Q. He Y. (2021b). *In silico* screening of circulating tumor DNA, circulating microRNAs, and long non-coding RNAs as diagnostic molecular biomarkers in ovarian cancer: a comprehensive meta-analysis. PLoS One 16 (4), e0250717. 10.1371/journal.pone.0250717 33901236 PMC8075214

[B195] Zhang L. Volinia S. Bonome T. Calin G. A. Greshock J. Yang N. (2008). Genomic and epigenetic alterations deregulate microRNA expression in human epithelial ovarian cancer. Proc. Natl. Acad. Sci. 105 (19), 7004–7009. 10.1073/pnas.0801615105 18458333 PMC2383982

[B196] Zhao L. Liang X. Wang L. Zhang X. (2022). The role of miRNA in ovarian cancer: an overview. Reprod. Sci. 29, 2760–2767. 10.1007/s43032-021-00717-w 34973152 PMC9537199

[B197] Zheng H. Liu J.-Y. Song F.-J. Chen K.-X. (2013a). Advances in circulating microRNAs as diagnostic and prognostic markers for ovarian cancer. Cancer Biol. Med. 10 (3), 123–130. 10.7497/j.issn.2095-3941.2013.03.001 24379986 PMC3860338

[B198] Zheng H. Zhang L. Zhao Y. Yang D. Song F. Wen Y. (2013b). Plasma miRNAs as diagnostic and prognostic biomarkers for ovarian cancer. PloS one 8 (11), e77853. 10.1371/journal.pone.0077853 24223734 PMC3815222

[B199] Zheng X. Chen S. Li L. Liu X. Liu X. Dai S. (2018). Evaluation of HE4 and TTR for diagnosis of ovarian cancer: comparison with CA-125. J. Gynecol. obstetrics Hum. reproduction 47 (6), 227–230. 10.1016/j.jogoh.2018.03.010 29609043

[B200] Zhou Q. Zuo M.-Z. He Z. Li H.-R. Li W. (2018). Identification of circulating microRNAs as diagnostic biomarkers for ovarian cancer: a pooled analysis of individual studies. Int. J. Biol. Markers 33 (4), 379–388. 10.1177/1724600818766500 29683066

